# Effects of gestational inflammation on age-related cognitive decline and hippocampal *Gdnf-GFRα1* levels in F1 and F2 generations of CD-1 Mice

**DOI:** 10.1186/s12868-023-00793-5

**Published:** 2023-04-13

**Authors:** Bao-Ling Luo, Zhe-Zhe Zhang, Jing Chen, Xue Liu, Yue-Ming Zhang, Qi-Gang Yang, Gui-Hai Chen

**Affiliations:** 1 Department of Neurology (Sleep Disorders), the Affiliated Chaohu Hospital of Anhui Medical University, Hefei, 238000 Anhui People’s Republic of China; 2 Department of Geriatrics, the First Affiliated Hospital of Bengbu Medical College, Bengbu, 233000 Anhui People’s Republic of China; 3 Department of Critical Care Medicine, the First Affiliated Hospital of Anhui Medical University, Hefei, 230022 Anhui People’s Republic of China

**Keywords:** Ageing, Learning and memory, GDNF-GFRα1, Inflammation, Intergenerational transmission, Mice

## Abstract

**Background:**

It has been reported that age-associated cognitive decline (AACD) accelerated by maternal lipopolysaccharide (LPS) insult during late pregnancy can be transmitted to the second generation in a sex-specificity manner. In turn, recent studies indicated that glial cell line‐derived neurotrophic factor (GDNF) and its cognate receptor (GFRα1) are critical for normal cognitive function. Based on this evidence, we aimed to explore whether *Gdnf-GFRα1* expression contributes to cognitive decline in the F1 and F2 generations of mouse dams exposed to lipopolysaccharide (LPS) during late gestation, and to evaluate also the potential interference effect of pro-inflammatory cytokines.

**Methods:**

During gestational days 15–17, pregnant CD-1 mice (8–10 weeks old) received a daily intraperitoneal injection of LPS (50 μg/kg) or saline (control). In utero LPS-exposed F1 generation mice were selectively mated to produce F2 generation mice. In F1 and F2 mice aged 3 and 15 months, the Morris water maze (MWM) was used to evaluated the spatial learning and memory ability, the western blotting and RT-PCR were used for analyses of hippocampal *Gdnf* and *GFRα1* expression, and ELISA was used to analyse IL-1β, IL-6 and TNF-α levels in serum.

**Results:**

Middle-aged F1 offspring from LPS-treated mothers exhibited longer swimming latency and distance during the learning phase, lower percentage swimming time and distance in targe quadrant during memory phase, and lower hippocampal levels of *Gdnf and GFRα1* gene products compared to age-matched controls. Similarly, the middle-aged F2 offspring from the Parents-LPS group had longer swimming latency and distance in the learning phase, and lower percentage swimming time and distance in memory phase than the F2-CON group. Moreover, the 3-month-old Parents-LPS and 15-month-old Parents- and Father-LPS groups had lower GDNF and GFRα1 protein and mRNAs levels compared to the age-matched F2-CON group. Furthermore, hippocampal levels of *Gdnf* and *GFRα1* were correlated with impaired cognitive performance in the Morris water maze after controlling for circulating pro-inflammatory cytokine levels.

**Conclusions:**

Our findings indicate that accelerated AACD by maternal LPS exposure can be transmitted across at least two generations through declined *Gdnf* and *GFRα1* expression, mainly via paternal linage.

**Supplementary Information:**

The online version contains supplementary material available at 10.1186/s12868-023-00793-5.

## Introduction

Age-associated cognitive decline (AACD) is associated with chronic low-grade inflammation, higher propensity to develop neuropsychiatric disorders, and death, and imposes a significant burden on individuals and society. Interestingly, epidemiological studies suggest that cognitive dysfunction can be transmitted across generations. Though mounting evidence indicates that AACD is associated with structural and functional alterations in the ageing hippocampus, including oxidative stress, inflammation, altered intracellular signalling and gene expression, as well as reduced neurogenesis and synaptic plasticity [1–4], the mechanisms underlying intergenerational inheritance of AACD remain unclear. Thus, additional research on this topic is needed to effectively prevent and treat cognitive dysfunction resulting from non-normative ageing in both parents and offspring.

Pregnant women are more susceptible to bacterial or viral infections and respond to them more strongly than non-pregnant women. In animal studies, bacterial-derived lipopolysaccharide (LPS) is commonly employed to mimic bacterial infections. Administration of LPS to pregnant dams leads to microglia and/or astrocyte activation and induces the expression of pro-inflammatory cytokines such as interleukin-1β (IL-1β), IL-6 and tumour necrosis factor-α (TNF-α). These can be passively transported into the foetal circulation, affecting the development of the fatal nervous system and compromising adult neurogenesis [5]. Moreover, LPS-induced systemic inflammation in gestating dams was shown to trigger a series of cellular and molecular events that can trigger neuroinflammation and affect, from infancy to adulthood, cognitive function in the offspring [6, 7]. The environmental influence on the neuronal epigenome is the greatest during the gestational period [8]. Notably, multiple sources of evidence have revealed that the deterioration of mental and behavioural capacity induced by prenatal and early life stressors can be inherited over multiple generations [9–12]. Although anxiety-like behaviour was detected in the offspring of both mothers and fathers subjected to neonatal LPS exposure, it was suggested that transmission of increased corticosterone activity only occurs in progeny of LPS-treated mothers [11]. This would indicate that there are differences in intergenerational inheritance patterns between maternal and paternal lineages. Our latest studies have showed that generational transfer of maladaptation on emotional and cognitive development in conditions of maternal LPS exposure could pass on to the second generation in a sex-dependent manner [13, 14]. In our previous work we have explored the role of epigenetic mechanisms in intergenerational transmission. However, other mechanisms are likely to be involved.

For instance, recent experiments on rats suffering from neuroinflammation induced by anaesthesia-surgery showed that impaired learning and memory is accompanied by decreased levels of glial cell line‐derived neurotrophic factor (GDNF) and reduced neurogenesis in the hippocampus [15]. GDNF, a member of the GDNF family ligands (GFLs), is widely expressed in multiple brain regions, including the striatum, hippocampus, cortex, and cerebellum [16]. GDNF signals through high-affinity binding with GDNF family receptor α1 (GFRα1), which is highly expressed in neurogenic areas of the postnatal brain. GDNF-GFRα1 signalling plays a crucial role in dendritic growth, morphological differentiation of dendritic arbours and spines, and synapse formation in hippocampal pyramidal neurons during early postnatal development [17, 18, 19,]. For example, Bonafina et al. demonstrated that GDNF signalling via GFRα1 mediates morphological maturation of hippocampal dentate gyrus granule cells and that GFRa1 deficiency results in impaired processing of spatial memory [17]. Moreover, GDNF exhibits a potent effect on the peripheral nervous system, influencing survival and growth of neurons in the somatic and autonomic nervous systems [20]. Numerous clinical and basic experimental studies have shown that the GDNF-GFRα1 complex plays an important role in hippocampal cognitive function [16, 21–23]. However, research so far has not addressed the association between intergenerational transmission of susceptibility to premature or accelerated AACD and altered hippocampal *Gdnf* and *GFRα1* expression.

Growing evidence shows that under immunologically non-challenged conditions, cytokines act as neuromodulators and facilitate normal learning and memory abilities [24, 25]. IL-1β, IL-6 and TNF-α are the most common pro-inflammatory cytokines; in inflammatory states (such as LPS challenge), these factors amplify their own production via autocrine induction and interact with other inflammatory mediators. A large body of literature indicates that over-expression of IL-1β, IL-6 and TNF-α has a detrimental effect on cognitive function [26–29] by affecting synaptic plasticity, long-term potentiation, neurogenesis, and brain structures (especially hippocampal regions) [30–33]. However, it remains unclear whether maternal gestational inflammation leads to altered GDNF levels and promotes AACD in direct (F1) and subsequent (F2) offspring.

To address this question, in the current study we measured the levels of IL-1β, IL-6 and TNF-α in F1 and F2 offspring from mouse dams treated with LPS during late pregnancy and analyzed their potential correlation with both cognitive function and hippocampal *Gdnf* and *GFRα1* expression. Our findings shed light on the molecular mechanisms underlying intergenerational transmission of predisposition to accelerated AACD following maternal gestational inflammation, and may help design novel therapeutic strategies to prevent or mitigate AACD in susceptible progeny.

## Results

### Performance of F1 and F2 mice in MWM test

#### Age effect

**Fig. 1:**
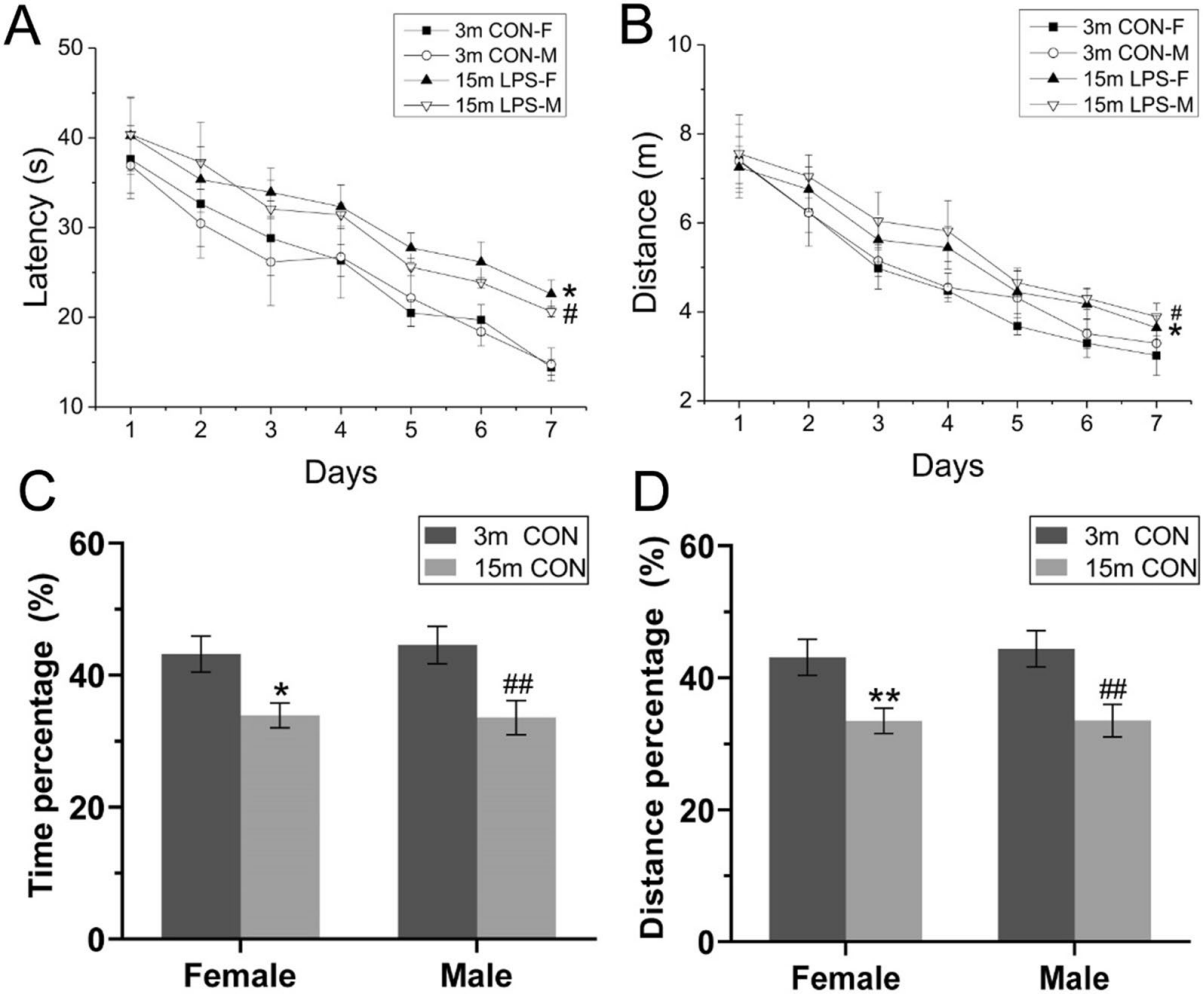
MWM performance in F1 generation in the control group at the age of 3 months (3 m) and 15 months (15 m). Swimming latency (**A**), and distance (**B**) during the learning phase. **C** Percentage swimming time and (**D**) percentage swimming distance during the memory phase. n = 10 per group. All data are presented as the mean ± SEM. * *P* < 0.05, ** *P* < 0.01 compared with female mice. ^#^*P* < 0.05, ^##^*P* < 0.01 compared with male mice. *CON-F* female mice exposed to saline in utero, *CON-M* male mice exposed to saline in utero, *LPS-F* female mice exposed to inflammation in utero, *LPS-M* male mice exposed to inflammation in utero

*Learning phase*. The swimming latency [*F*_(6, 216)_ = 26.650, *P* < 0.01; Fig. 1A] and distance [*F*_(6, 216)_ = 35.968, P < 0.01; Fig. 1B] progressively declined over days for all CON mice, indicating that the mice had the ability to learn the task. Among these, and reflecting an expected age-related effect, two-way repeated measures ANOVA indicated significantly longer swimming latency [*F*_(1, 36)_ = 10.873, *P* < 0.01] and distance [*F*_(1, 36)_ = 10.949, *P* < 0.01] for 15-months-old compared to 3-months-old mice. Regarding swimming velocity, no significant differences were observed between the two groups [*F*_(1, 38)_ = 1.973, *P* = 0.168; Additional file 1A;]. The effects of sex and interactions of age × sex, age × day, sex × day, and age × sex × day were non-significant in the learning phase of the trials.

*Memory phase*. Two-way ANOVA revealed for 15-months-old CON mice a significantly decreased percentage swimming time [*F*_(1, 36)_ = 16.10, *P* < 0.01; Fig. 1C] and swimming distance [*F*_(1, 36)_ = 16.95, *P* < 0.01; Fig. 1D] in the target quadrant compared to 3-months-old CON mice, for both sexes. No sex difference or interaction of age × sex effect was observed in the memory phase.

#### Treatment effect

**Fig. 2:**
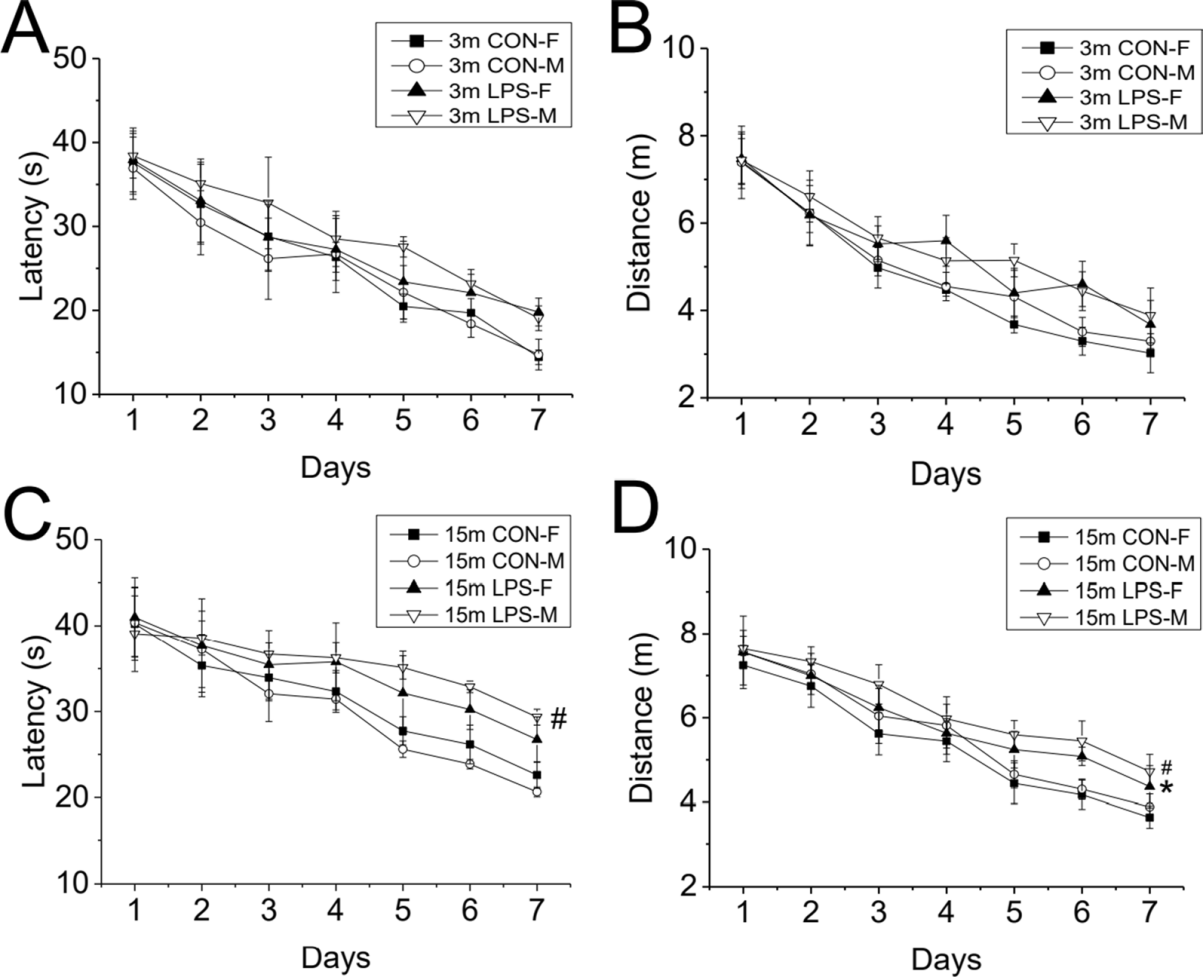
Learning performance in the MWM test for the F1 generation at 3 months (3 m) and 15 (15 m) months of age. Escape latency (**A**, **C**) and swimming distance (**B**, **D**) during the learning phase. n = 10 per group. Data are presented as the mean ± SEM. *P < 0.05 compared with CON-F. ^#^*P* < 0.05 compared with CON-M. *CON-F* female mice exposed to saline in utero, *CON-M* male mice exposed to saline in utero, *LPS-F* female mice exposed to inflammation in utero, *LPS-M* male mice exposed to inflammation in utero

*Learning phase*. In the F1 generation, LPS-treatment effects on mice of 3 months of age were significant on swimming distance [*F*_(1, 36)_ = 6.016, *P* = 0.019], with longer swimming distance exhibited by the LPS group, but not on swimming latency [*F*_(1, 36)_ = 2.816, *P* = 0.102] [Fig. 2A, B]. However, the post hoc analyses showed that there was no significant difference in the swimming distance between LPS and CON groups for females and males (*Ps* > 0.05). At 15 months of age, a longer swimming latency and distance was recorded for F1 mice from LPS-treated mothers relative to CON mice [*F*_(1, 36)_ = 5.275, 9.925; *P* = 0.028, *P* < 0.01; Fig. 2C, D]. Post-hoc analyses showed that male offspring from LPS-treated mothers (LPS-M) had significant longer swimming latency and distance than CON-M mice [*P* = 0.045, 0.025], whereas a longer swimming distance (*P* = 0.042), but comparable swimming latency, was observed for female offspring from LPS-treated mothers (LPS-F) compared to the CON-F group. In turn, there were no significant differences in swimming velocity between mice in the LPS and CON groups at both 3 months [*F*_(1, 38)_ = 0.138, P = 0.712; Additional file 1B;] and 15 months [*F*_(1, 38)_ = 0.152, P = 0.699; Additional file 1C;]. No sex difference or interactions of group × sex, group × day, sex × day or group × sex × day were observed at either 3 or 15 months of age.

**Fig. 3:**
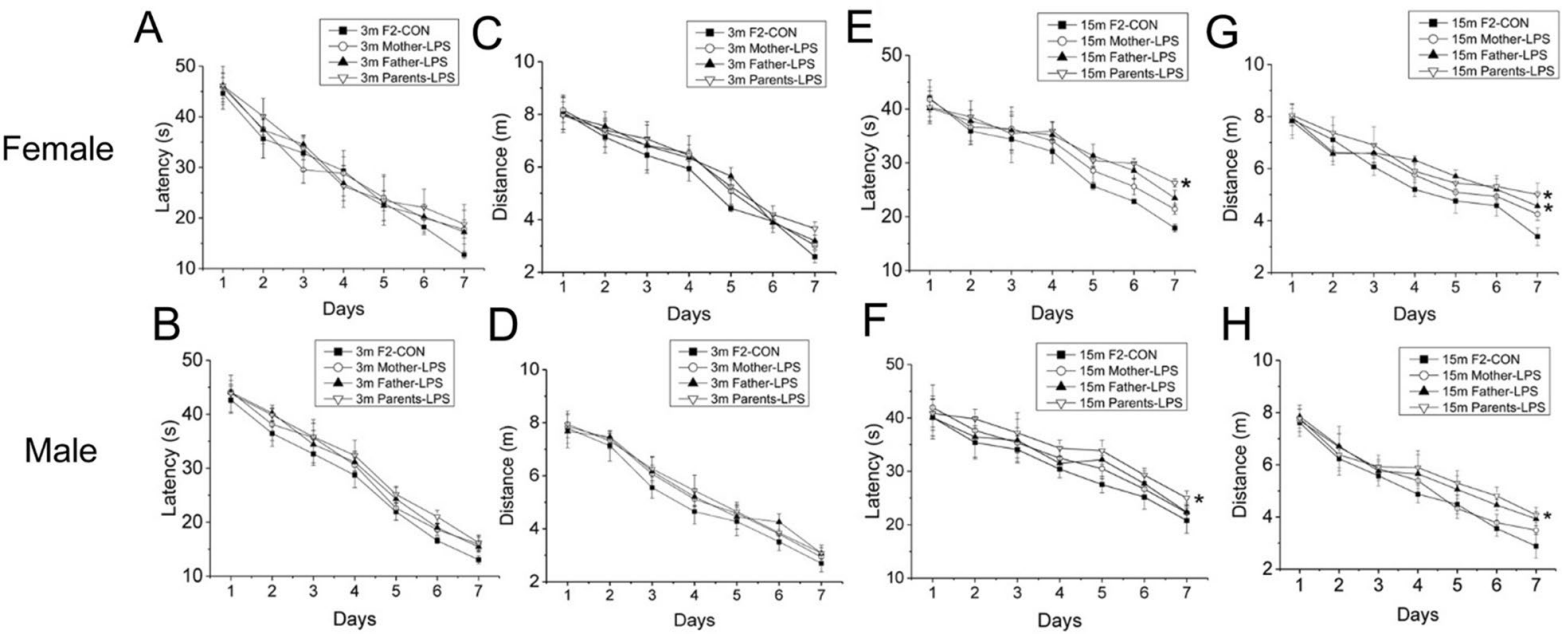
Learning performance in the MWM test for the F2 generation. Escape latency (**A**, **B**, **E**, **F**) and swimming distance (**C**, **D**, **G**, **H**) for 3-months-old (3 m) and 15-months-old (15 m) CD-1 mice. Data are depicted as the mean ± SEM. n = 10 per group. *P < 0.05 compared with F2-CON. *F2-CON* mice whose parents were exposed to saline in utero, *Mother-LPS* mice whose mothers were exposed to inflammation in utero, *Father-LPS* mice whose fathers were exposed to inflammation in utero, *Parents-LPS* mice whose parents were exposed to inflammation in utero

In the F2 generation, 3-months-old mice exhibited no LPS-related differences in swimming latency [*F*_(3, 72)_ = 1.271, *P* = 0.291; Fig. 3A, B], swimming distance [*F*_(3, 72)_ = 2.096, *P* = 0.108; Fig. 3C, D] and swimming velocity [*F*_(3, 76)_ = 0.243, P = 0.866; Additional file 1D;]. There was, however, a significant effect of sex on distance swam [*F*_(1, 72)_ = 10.358, *P* < 0.01] among the four groups (i.e. F2-CON, Mother-LPS, Father-LPS and Parents-LPS). At 15 months of age, F2 mice born from LPS-exposed parents showed longer swimming latency [F _(3, 72)_ = 3.608, *P* = 0.017; Fig. 3E, F] and distance [*F*_(3, 72)_ = 5.191, *P* < 0.01; Fig. 3G, H] compared to the F2-CON group, whereas no significant difference in swimming velocity [*F*_(3, 76)_ = 0.942, *P* = 0.424; Additional file 1E;] was detected among the four groups. Meanwhile, a significant effect of sex was observed in distance swam [*F*_(1, 72)_ = 15.093, *P* < 0.01], with Mother-LPS females achieving a longer mean swimming distance than counterpart males (*P* = 0.042). Moreover, for both sexes, 15-months-old mice in the Parents-LPS group displayed longer swimming latency and distance than the F2-CON group (*Ps* < 0.05), while Father-LPS male mice exhibited a longer distance swam compared to F2-CON male mice (*P* = 0.033). No significant interactions of group × sex, group × day, sex × day, and group × sex × day were observed during the learning phase in mice of 3 and 15 months of age.

**Fig. 4:**
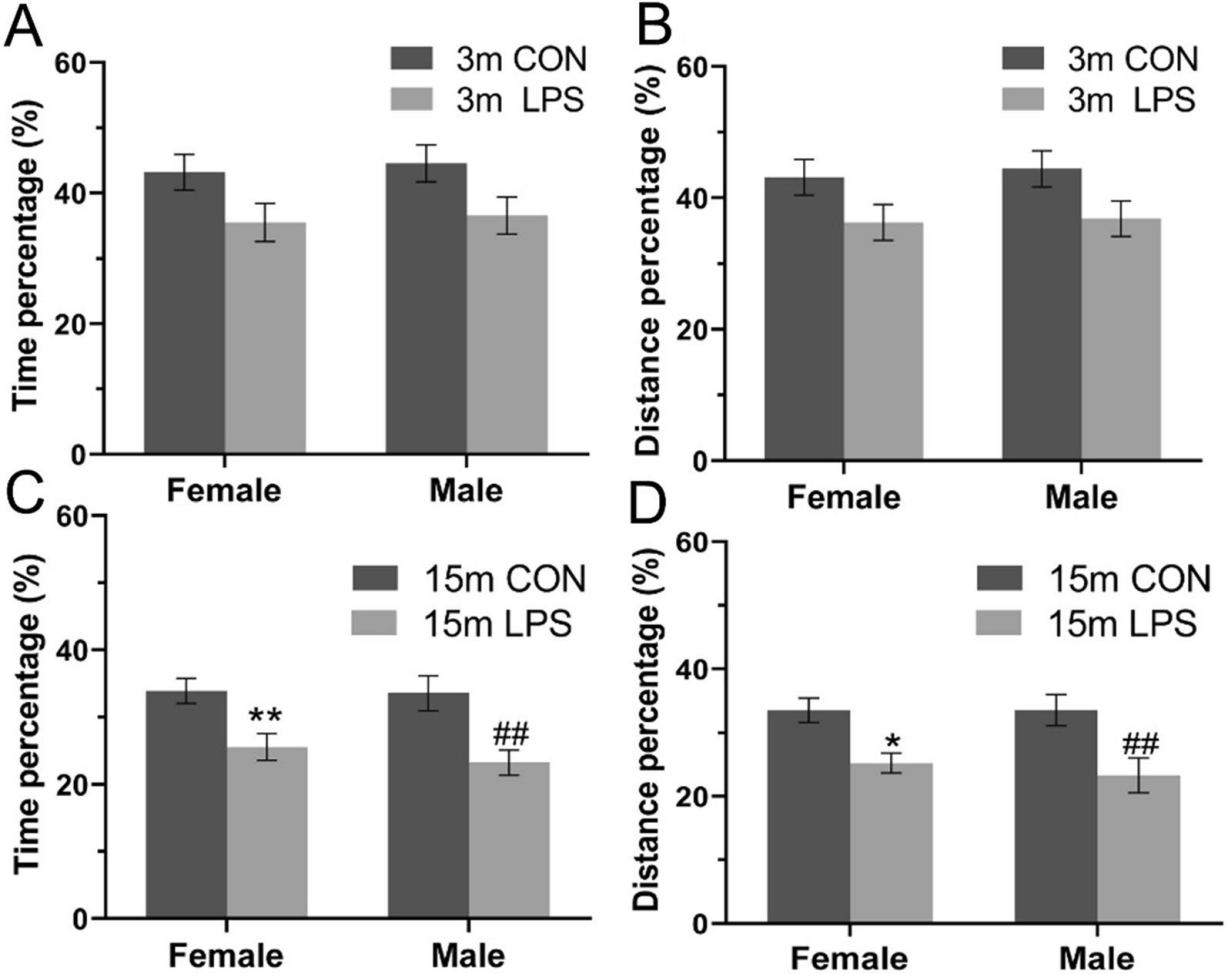
Memory performance in the MWM test for the F1 generation at 3 months (3 m) and 15 months (15 m) of age. Percentage swimming time (**A**, **C**) and percentage swimming distance (**B**, **D**) during the memory phase. n = 10 per group. Data are presented as the mean ± SEM. * *P* < 0.05, ** *P* < 0.01 compared with female mice in control group. ^##^*P* < 0.01 compared with male mice in control group. *CON* mice exposed to saline in utero, *LPS* mice exposed to inflammation in utero

*Memory phase*. In 3-months-old mice of both sexes in the F1 generation, LPS treatment significantly affected percentage swimming time [*F*_(1. 36)_ = 7.802, *P* < 0.01; Fig. 4A] and swimming distance [*F*_(1. 36)_ = 8.123, *P* < 0.01; Fig. 4B] in the target quadrant, with lower percentage of swimming time and distance being observed in the LPS groups. However, the post hoc analyses indicated that no significant difference was observed in percentage swimming time and distance between LPS and CON groups for females and males (*Ps* > 0.05). At 15 months of age, LPS-treated mice exhibited decreased percentage swimming time and distance than CON mice for the combined sexes [*F*_(1. 36)_ = 19.89, 17.48; *P* < 0.01, < 0.01; Fig. 4C, D]. The effects of sex and interaction of group × sex were non-significant in the memory phase at 3 and 15 months of age.

**Fig. 5:**
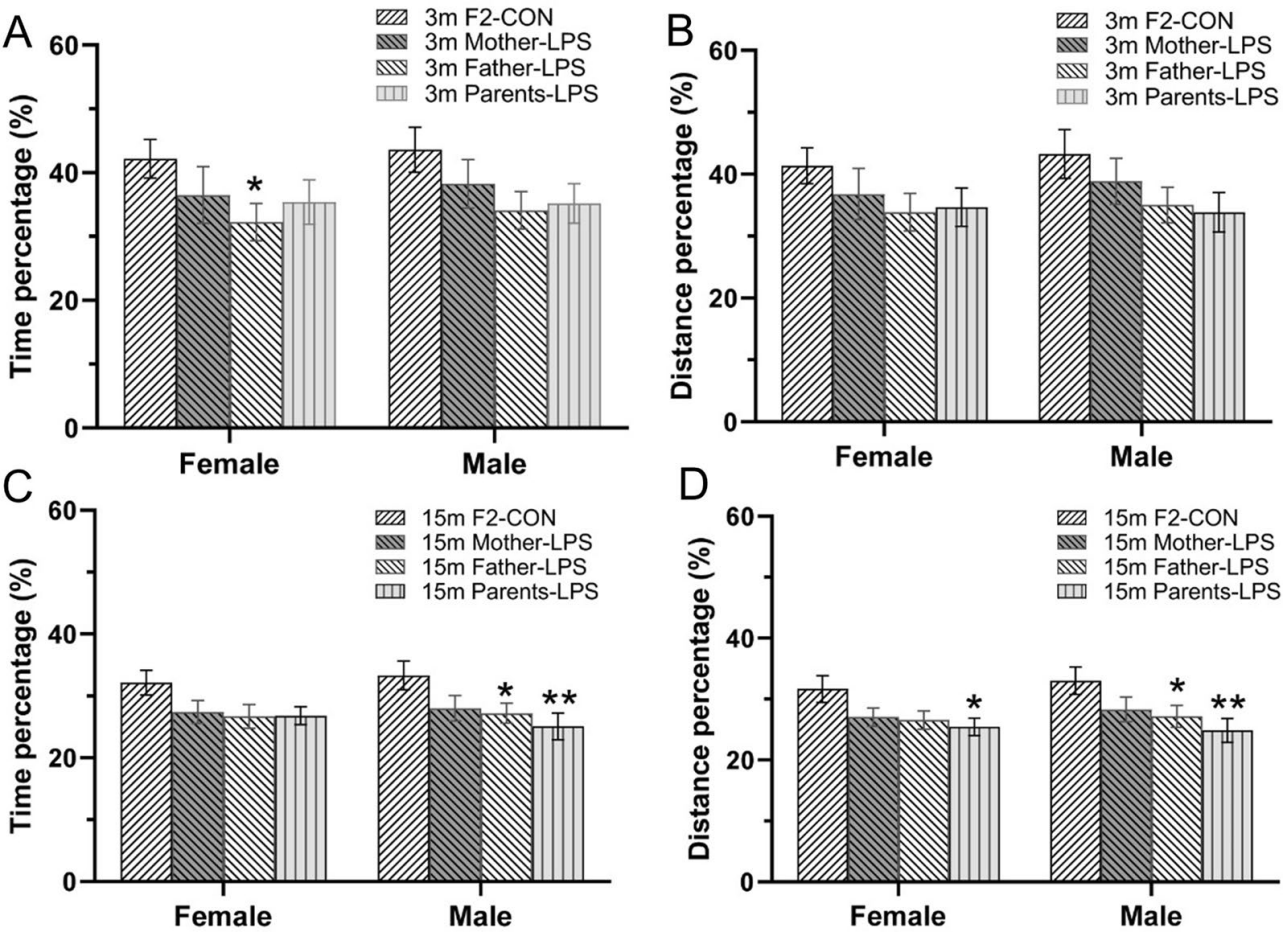
Memory performance in the MWM test for the F2 generation. Percentage swimming time (**A**, **C**) and percentage swimming distance (**B**, **D**) during the memory phase for 3-months-old (3 m) and 15-month-old (15 m) CD-1 mice. n = 10 per group. Data are expressed as the mean ± SEM.* *P* < 0.05, ** *P* < 0.01 compared with F2-CON. *F2-CON* mice whose parents were exposed to saline in utero, *Mother-LPS* mice whose mothers were exposed to inflammation in utero, *Father-LPS* mice whose fathers were exposed to inflammation in utero, *Parents-LPS* mice whose parents were exposed to inflammation in utero

In the F2 generation, 3-months-old mice from parents exposed to LPS showed reduced percentage of swimming time [*F*_(3, 72)_ = 2.933, *P* = 0.039; Fig. 5A] compared to the F2-CON group, with differences between groups derived primarily from Father-LPS females (P = 0.04). There were in turn no significant differences on percentage of swimming distance [*F*_(3, 72)_ = 2.482, *P* = 0.068; Fig. 5B] among CON and LPS groups. In mice aged 15 months, both percentage of swimming time [*F*_(3, 72)_ = 4.832, *P* < 0.01; Fig. 5C] and distance [*F*_(3, 72)_ = 5.463, *P* < 0.01; Fig. 5D] were decreased in the LPS groups relative to the F2-CON group. Post-hoc analyses showed that such differences arose primarily from the Father-LPS and Parents-LPS groups. Neither sex nor the interactions involving group × sex had significant effects during the memory phase in 3- and 15-months-old mice.

### Hippocampal expression of *Gdnf* and *GFRα1* in F1 and F2 mice

#### GDNF and GFRα1 protein levels

##### Age effect

**Fig. 6:**
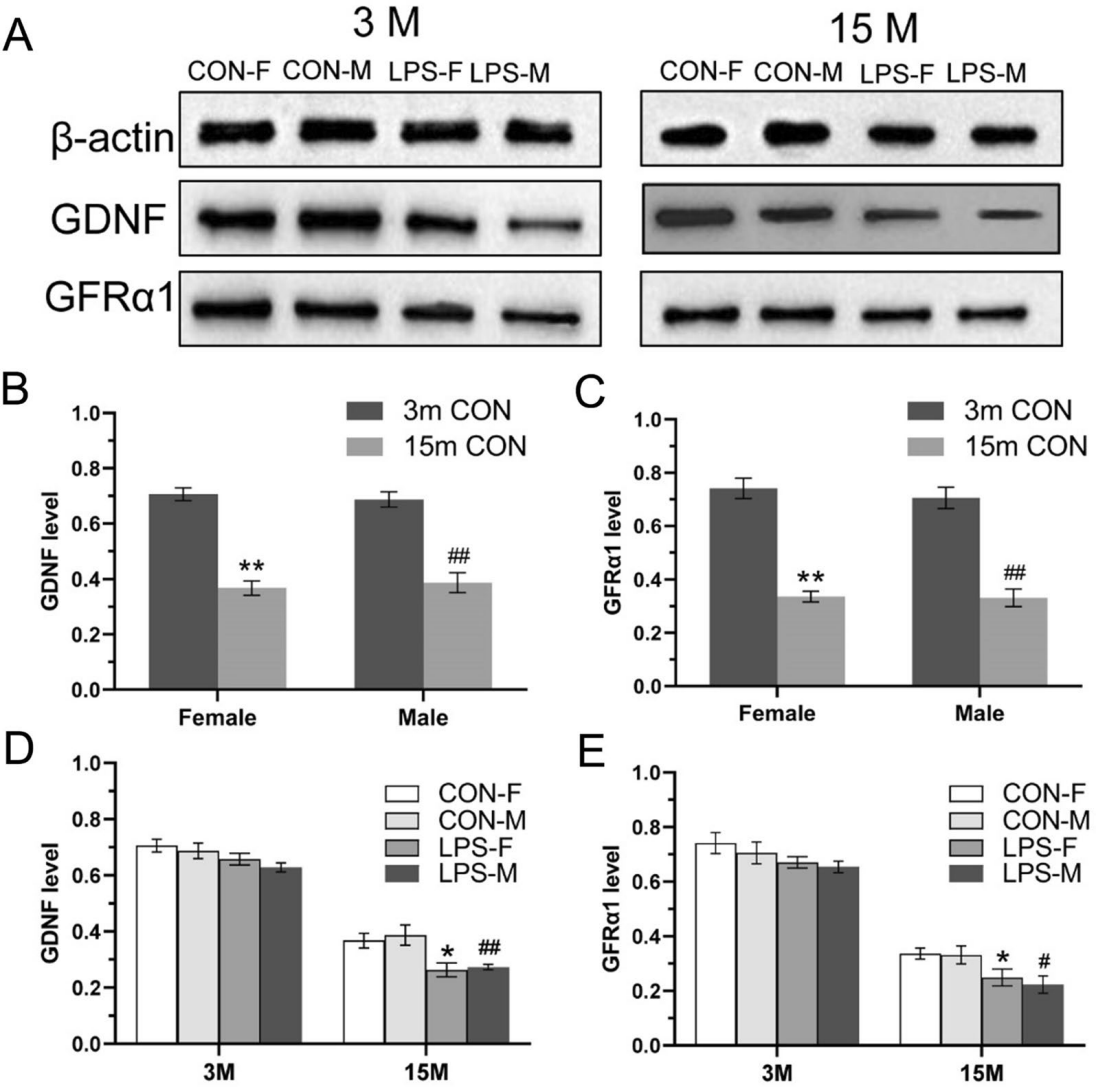
Analysis of GDNF and GFRα1 protein expression in the hippocampus of F1 mice. **A**: cropped representative immunoreactive bands for GDNF and GFRα1 in the hippocampi of F1 mice aged 3 months (3 M) and 15 months (15 M). **B, C**: quantification of GDNF (**B**) and GFRα1 (**C**) protein levels in CON mice. **D**, **E**: Quantification of GDNF and GFRα1 protein levels across treatments and ages. n = 6 per group. Data are presented as mean ± SEM. * *P* < 0.05, ** *P* < 0.01 compared with CON-F. ^#^*P* < 0.05, ^##^*P* < 0.01 compared with CON-M. *CON-F* female mice exposed to saline in utero, *CON-M* male mice exposed to saline in utero, *LPS-F* female mice exposed to inflammation in utero, *LPS-M* male mice exposed to inflammation in utero

Cropped representative GDNF and GFRα1 immunoreactive bands in the hippocampi of F1 mice at 3- and 15-month-old are shown in Fig. 6A and the full-length blot was seen in Additional file 2. Two-way ANOVA showed that 15-months-old CON mice had lower GDNF and GFRα1 protein expression relative to 3-months-old mice for the combined sexes [*F*_(1, 20)_ = 125.8, 133.3; *P* < 0.01, < 0.01; Fig. 6B, C]. There was in turn no significant sex effect or interaction of sex × age.

##### Treatment effect

In F1 mice of 3 months of age, lower levels of hippocampal GDNF protein were observed in the LPS-treated group compared with the CON group for the combined sexes [*F*_(3, 72)_ = 5.854, *P* = 0.025; Fig. 6D], but not for the females or males (*Ps* > 0.05). There was no major difference in GFRα1 protein level between the LPS-exposure group and the control group [*F*_(3, 72)_ = 3.780, *P* = 0.066; Fig. 6E]. At 15 months of age, for the combined sexes protein levels of GDNF and GFRα1 in the LPS group were significantly lower than those in the CON group [*F*_(1, 20)_ = 17.68, 11.20; *P* < 0.01, < 0.01; Fig. 6D, E]. Neither the effect of sex nor the interaction of group × sex significantly affected GDNF and GFRα1 levels at both 3 and 15 months of age.

**Fig. 7:**
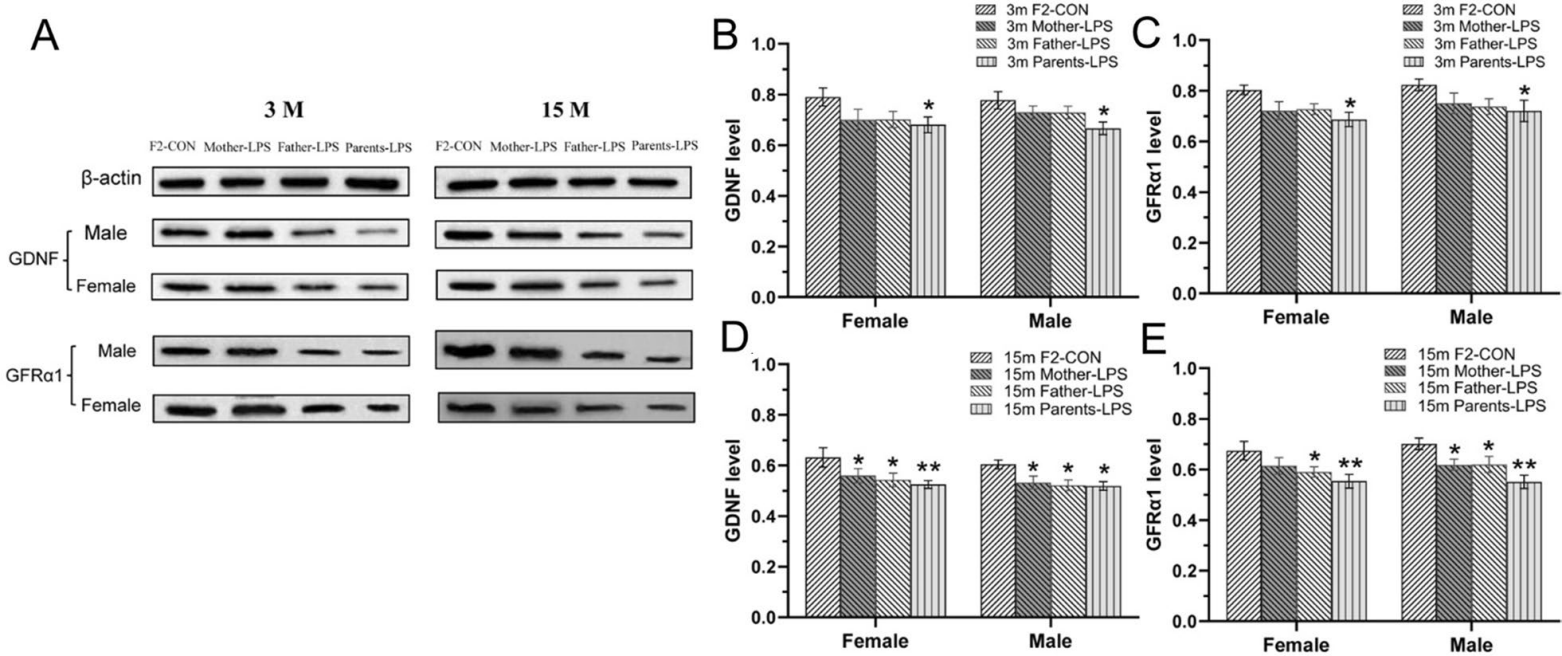
Analysis of GDNF and GFRα1 protein expression in the hippocampus of F2 mice. **A**: cropped representative immunoreactive bands for GDNF and GFRα1 across treatments and ages. **B-E**: quantification of GDNF (**B**, **D**) and GFRα1 (**C**, **E**) protein levels across treatments in mice of 3 months (3 m) and 15 months (15 m) of age. n = 6 per group. Data are presented as mean ± SEM. * *P* < 0.05, ** *P* < 0.01 compared with F2-CON group. *F2-CON* mice whose parents were exposed to saline in utero, *Mother-LPS* mice whose mothers were exposed to inflammation in utero, *Father-LPS* mice whose fathers were exposed to inflammation in utero, *Parents-LPS* mice whose parents were exposed to inflammation in utero

Cropped representative GDNF and GFRα1 immunoreactive bands in the hippocampi of F2 mice at 3- and 15-month-old are shown in Fig. 7A and the full-length blot was seen in Additional file 3. At 3 months of age, and relative to the F2-CON group, lower levels of hippocampal GDNF [*F*_(3, 40)_ = 4.171, *P* = 0.012; Fig. 7B] and GFRα1 [*F*_(3, 40)_ = 4.553, *P* < 0.01; Fig. 7C] protein were noted in the LPS groups, particularly in the Parents-LPS group. At 15 months of age, two-way ANOVA indicated that mice in the LPS groups had lower levels of GDNF [*F*_(3, 40)_ = 6.136, *P* < 0.01; Fig. 7D] and GFRα1 [*F*_(3, 40)_ = 7.985, *P* < 0.01; Fig. 7E] protein than F2-CON mice, with pairwise comparisons indicating comparable reductions in GDNF and GFRα1 protein levels in the Mother-LPS, Father-LPS and Parents-LPS groups. Effects of sex and interaction of group × sex were not significant at both 3 and 15 months of age.

#### Gdnf and GFRα1 mRNAs levels

##### Age effect

**Fig. 8:**
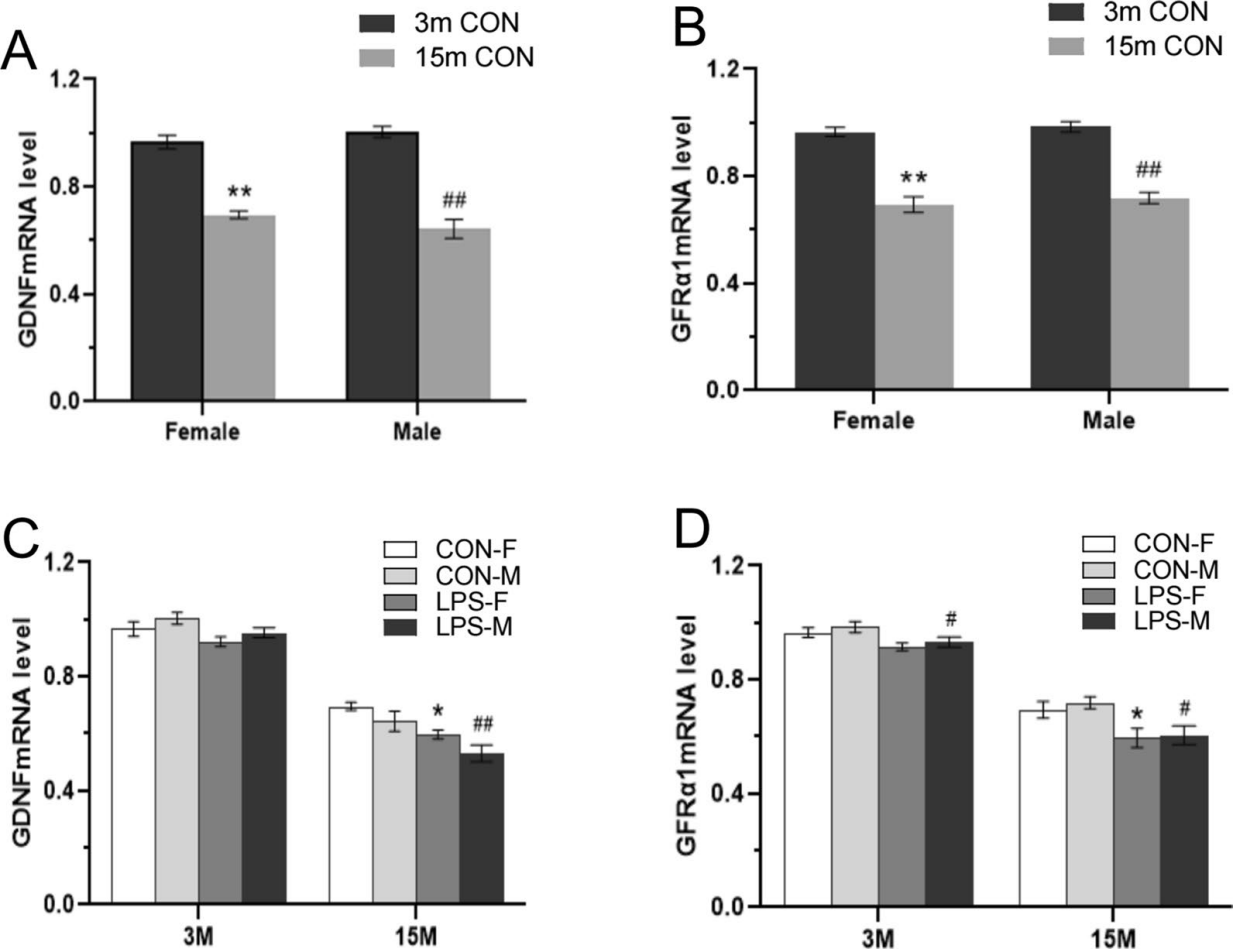
Analysis of *Gdnf* and *GFRα1* mRNAs expression in the hippocampus of F1 mice. **A**, **B**: relative* Gdnf* (A) and *GFRα1* (B) mRNAs expression levels in 3- and 15- months-old mice in the CON group. **C**, **D**: relative* Gdnf* (C) and *GFRα1* (D) mRNAs expression levels across treatments and ages. n = 6 per group. Data are presented as mean ± SEM. * *P* < 0.05, ** *P* < 0.01 compared with CON-F. ^#^*P* < 0.05, ^##^*P* < 0.01 compared with CON-M. *CON-F* female mice exposed to saline in utero, *CON-M* male mice exposed to saline in utero, *LPS-F* female mice exposed to inflammation in utero, *LPS-M* male mice exposed to inflammation in utero

As shown in Fig. 8A, B, relative to CON mice lower hippocampal *Gdnf* and *GFRα1* mRNAs levels were detected in 15-months-old mice compared to 3-months-old mice for the combined sexes [*F*_(1, 20)_ = 156.9, 145.6; *P* < 0.01, < 0.01], the females and males (*Ps* < 0.01). No effect of sex or interaction of age × sex was observed.

##### Treatment effect

In F1 mice aged 3 months, lower *Gdnf* and *GFRα1* mRNAs levels were detected in hippocampi from the LPS groups, relative to the CON group for both sexes [*F*_(1, 20)_ = 5.30, 9.148; *P* = 0.0322, *P* < 0.01; Fig. 8C, D]. However, the post hoc analyses showed that only the LPS-M group had significantly lower *GFRα1* mRNA levels than the CON-M group (*P* < 0.05). No significant effect of sex on *Gdnf* and *GFRα1* mRNAs expression was established for these animals. In mice aged 15 months, LPS-treatment was associated with significant reductions in the levels of *Gdnf* and *GFRα1* mRNAs for the combined sexes [*F*_(1, 20)_ = 17.31, 13.01; *P* < 0.01, < 0.01; Fig. 8C, D], the females and the males (*Ps* < 0.05). A significant sex difference was noted in *Gdnf* mRNA [*F*_(1, 20)_ = 5.432, *P* = 0.030] but not in *GFRα1* mRNA expression [*F*_(1, 20)_ = 0.335, *P* = 0.5690], with females showing lower *Gdnf* mRNA level than males. In contrast, the interaction of group × sex was non-significant at both 3 and 15 months of age.

**Fig. 9:**
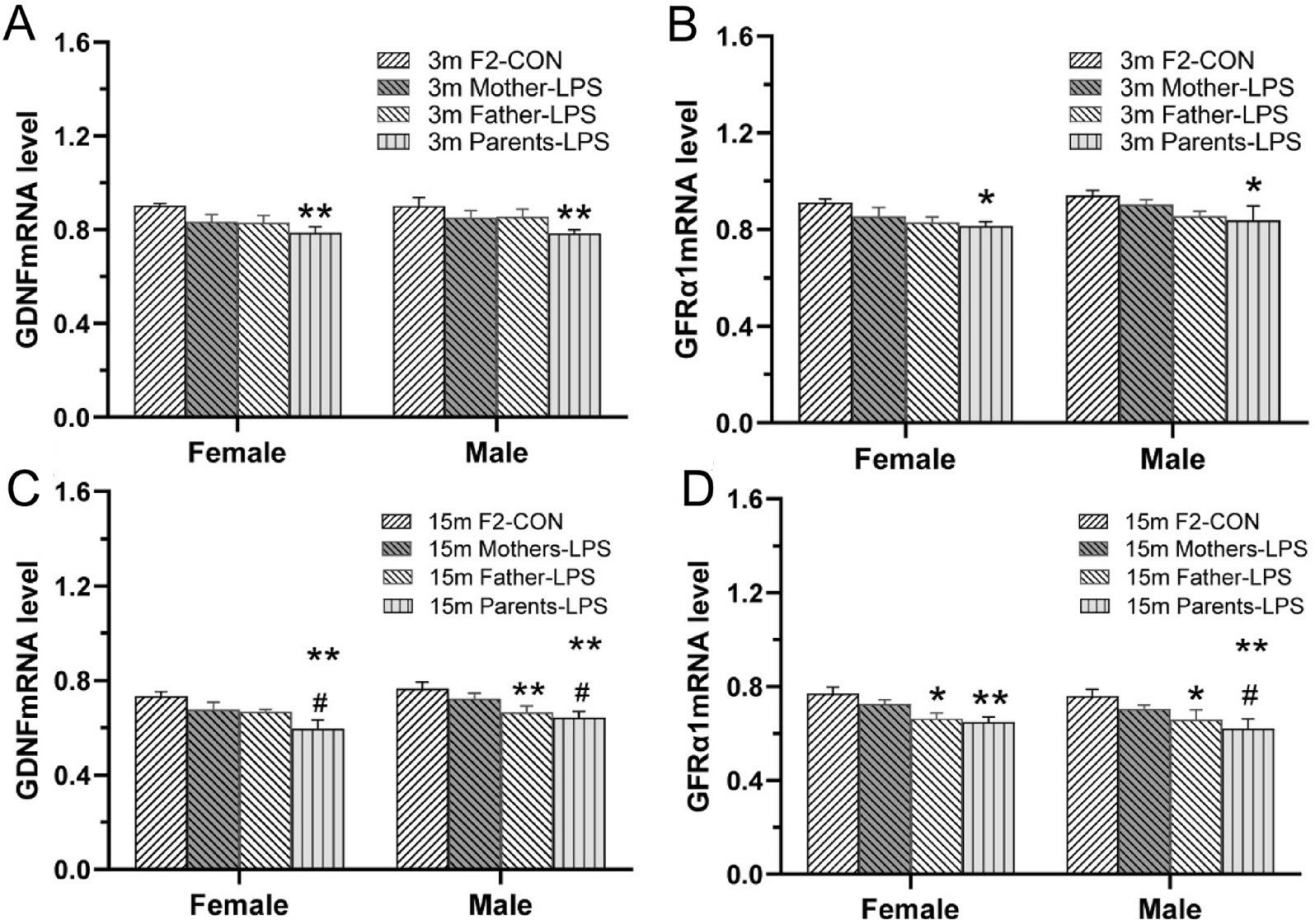
Analysis of *Gdnf* and *GFRα1* mRNAs expression in the hippocampus of F2 mice. **A-D:** quantification of *Gdnf* (**A**, **C**) and *GFRα1* (**B**, **D**) mRNAs levels in 3-months-old (3 m) (**A**, **B**) and 15-month-old (15 m) (**C**, **D**) mice from the different groups. n = 6 per group. Data are presented as mean ± SEM. *P < 0.05, **P < 0.01 compared with F2-CON. *F2-CON* mice whose parents were exposed to saline, *Mother-LPS* mice whose mothers were exposed to inflammation in utero, *Father-LPS*, mice whose fathers were exposed to inflammation in utero, *Parents-LPS* mice whose parents were exposed to inflammation in utero

For 3-months-old mice in the F2 generation, two-way ANOVA revealed that the Parents-LPS group had lower mRNAs levels of *Gdnf* and *GFRα1* than the F2-CON group for the combined sexes [*F*_(3, 40)_ = 6.173, 4.407; *P* < 0.01, < 0.01; Fig. 9A, B]. In turn, analysis of 15-months-old F2 mice showed that the LPS-exposed groups had lower *Gdnf* [*F*_(3, 40)_ = 8.824, *P* < 0.01; Fig. 9C] and *GFRα1* [*F*_(3, 40)_ = 8.267, *P* < 0.01; Fig. 9D] mRNAs levels compared to the F2-CON group. Post-hoc analyses showed that these effects were mainly derived from the Father-LPS and Parents-LPS groups (*Ps* < 0.05). Moreover, the Parents-LPS group showed lower levels of hippocampal *Gdnf* and *GFRα1* mRNAs relative to the Mother-LPS group (*Ps* < 0.05). No sex effect or interaction of group × sex was detected in 3- and 15-months-old mice.

### Assessment of serum IL-1β, IL-6 and TNF-α levels in F1 and F2 mice

#### Age effect

**Fig. 10:**
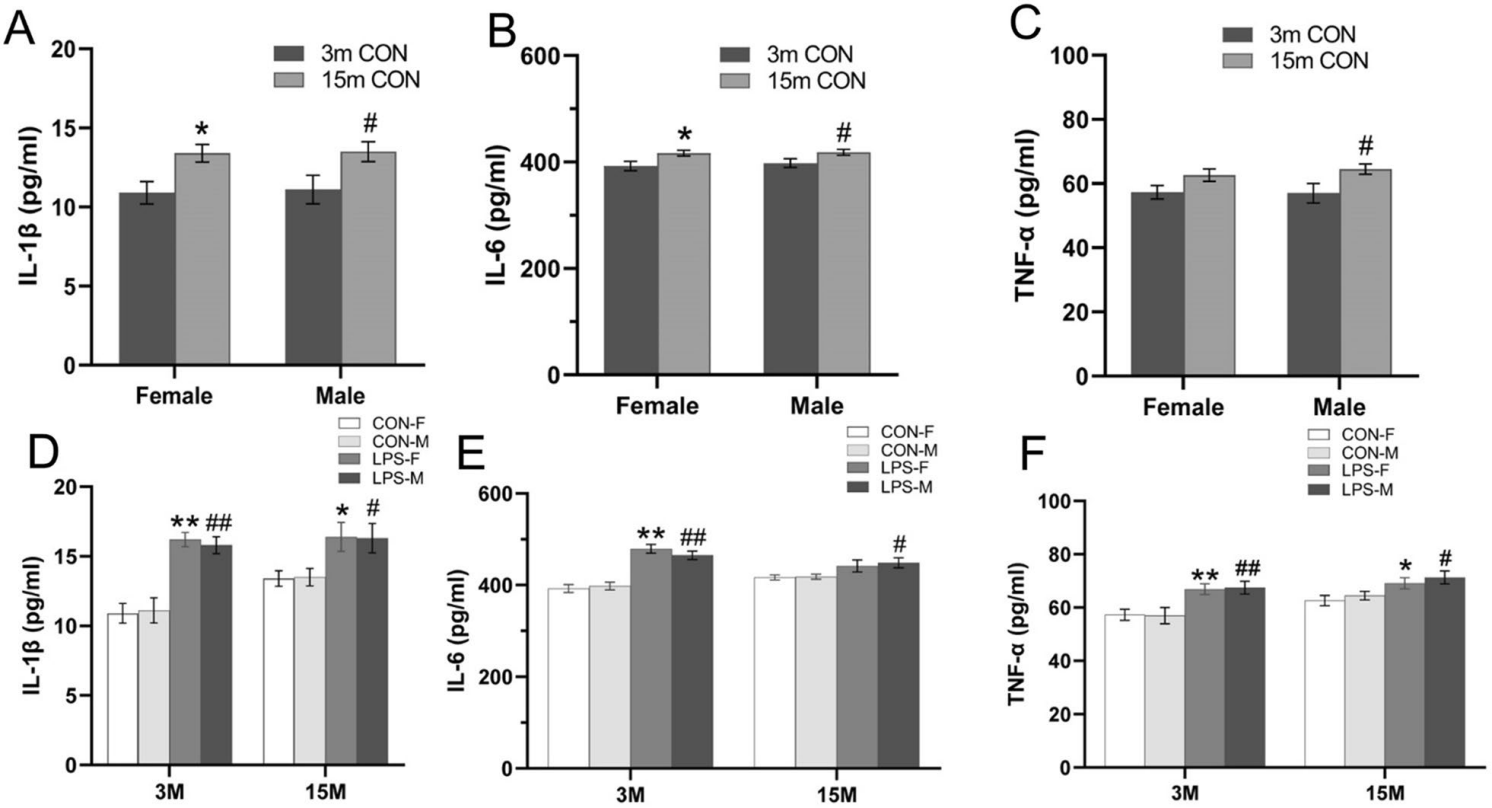
Serum levels of IL-6, TNF-α and IL-1β in F1 generation. **A-C:** serum levels of IL-1β (A), IL-6 (B) and TNF-α (C) in CON mice aged 3 months (3 m) and 15 months (15 m). **D-F:** serum IL-1β (D), IL-6 (E) and TNF-α (F) levels in mice from LPS and CON groups. n = 10 per group. Data are presented as mean ± SEM. *P < 0.05, **P < 0.01 compared to CON-F; ^#^*P* < 0.05, ^##^*P* < 0.01 compared to CON-M; *CON-F* female mice exposed to saline in utero, *CON-M* male mice exposed to saline in utero, *LPS-F*, female mice exposed to inflammation in utero, *LPS-M* male mice exposed to inflammation in utero

ELISA-based analyses on F1 CON animals indicated that irrespective of sex, 15-month-old mice had higher serum levels of both IL-1β [*F*_(1, 36)_ = 11.96, *P* < 0.01; Fig. 10A] and IL-6 [*F*_(1, 36)_ = 9.790, *P* < 0.01; Fig. 10B] than 3-month-old mice. In turn, higher TNF-α levels [*F*_(1, 36)_ = 8.098, *P* < 0.01; Fig. 10C] were detected in older male, but not in female mice.

#### Treatment effect

At both 3 and 15 months of age, male and female F1 mice from the LPS-treated groups exhibited higher serum IL-1β [*F*_(1, 36)_ = 51.43,11.67; *P* < 0.01, < 0.01; Fig. 10D] and TNF-α levels [*F*_(1, 36)_ = 17.16, 10.53; *P* < 0.01, < 0.01; Fig. 10F] than CON mice. Compared to the respective CON groups, higher levels of IL-6 were detected in 3-month-old LPS-F and LPS-M groups, as well as in 15-month-old LPS-M [*Ps* < 0.01; Fig. 10E]. For the three cytokines, no effects of sex or interaction between sex and groups were noted for 3- and 15-months-old mice (*Ps* > 0.05).

**Fig. 11:**
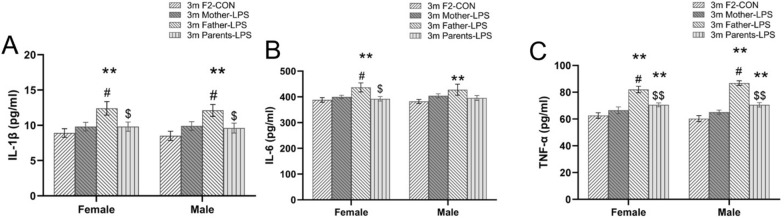
serum levels of IL-1β, IL-6 and TNF-α in F2 generation. **A-C**: serum levels of IL-1β (**A**), IL-6 (**B**) and TNF-α (**C**) for 3-months-old male and female F2 mice in the CON and LPS-treated groups. n = 10 per group. Data are presented as mean ± SEM. ** *P* < 0.01 compared with F2-CON; ^#^*P* < 0.05 compared with Mother-LPS; ^$^*P* < 0.05*,* ^$$^*P* < 0.01 compared with Father-LPS. *F2-CON* mice whose parents were exposed to saline in utero, *Mother-LPS* mice whose mothers were exposed to inflammation in utero, *Father-LPS* mice whose fathers were exposed to inflammation in utero, *Parents-LPS* mice whose parents were exposed to inflammation in utero

In F2 mice of 3 months of age, there were significant differences in the levels of IL-1β [*F*_(3, 72)_ = 8.844, *P* < 0.01; Fig. 11A], IL-6 [*F*_(3, 72)_ = 5.614, *P* < 0.01; Fig. 11B] and TNF-α [*F*_(3, 72)_ = 50.37, *P* < 0.01; Fig. 11C] among the four groups. Father-LPS mice exhibited higher IL-1β, IL-6 and TNF-α levels when compared to F2-CON and Parents-LPS groups (*Ps* < 0.05). Moreover, TNF-α levels in the Parents-LPS group were higher than in the F2-CON group for both sexes (*Ps* < 0.05). In contrast, at 15 months of age maternal/paternal LPS exposure had no effect on these cytokines (data not shown). No significant sex difference or interaction of group × sex effects were found in these analyses.

### Correlation between performance in MWM and *GDNF and GFRα1* protein and mRNAs levels

Results of Pearson’s correlation analyses between performance in MWM and *Gdnf* and *GFRα1* expression in mouse hippocampus are shown in Tables 1 and 2. In F1 generation, no correlations between cognitive performance and the GDNF and GFRα1 protein and mRNAs levels were found among groups of 3-months-old mice. However, for 15-months-old mice in the LPS-treatment groups, both GDNF and GFRα1 protein and mRNAs levels were negatively correlated with swimming distance in the learning phase and positively with the distance swam during the memory phase (*P*s < 0.05; Table 1). Meanwhile, in F2 mice of 3 months of age, GDNF protein expression was negatively correlated with distance swam in the Father-LPS group (*r* = − 0.626, *P* = 0.029). Also, among 3-months-old mice, *Gdnf* mRNA expression was negatively correlated with the distance swam in the learning phase (*r* = − 0.629, *P* = 0.029) and positively correlated with percent distance swam in the memory phase (*r* = 0.675, *P* = 0.016) in the Parents-LPS group. At 15 months of age, mRNAs levels of *Gdnf* and *GFRα1* were negatively correlated with the distance swam (*P*s < 0.05), and positively correlated with the percentage of distance swam in the Father-LPS and Parents-LPS groups (*P*s < 0.05; Table 2).

**Table 1:** The correlation between the performance in MWM and hippocampal GDNF-GFRα1 expression in F1 generation

Ages	Cognitive parameters	Groups	GDNF [r (p)]		GFRα1 [r (p)]	
			Protein	mRNA	Protein	mRNA
3 months	Swam distance	CON	− 0.482 (0.112)	− 0.572 (0.052)	− 0.388 (0.212)	− 0.342 (0.276)
		LPS	0.088 (0.785)	− 0.401 (0.196)	− 0.159 (0.621)	− 0.389 (0.211)
	Percentage swam distance in target quadrant	CON	0.450 (0.143)	0.536 (0.072)	0.500 (0.098)	0.507 (0.093)
		LPS	0.400 (0.197)	0.364 (0.245)	0.245 (0.444)	0.439 (0.153)
15 months	Swam distance	CON	− 0.519 (0.084)	− 0.554 (0.062)	− 0.524 (0.080)	− 0.290 (0.361)
		LPS	− 0.893 (0.000)**	− 0.788 (0.002)**	− 0.702 (0.011)*	− 0.788 (0.002)**
	Percentage swam distance in target quadrant	CON	0.099 (0.760)	0.146 (0.652)	0.230 (0.472)	0.167 (0.604)
		LPS	0.666 (0.018)*	0.799 (0.002)**	0.709 (0.010)**	0.776 (0.003)**

n = 6 per group. *P < 0.05, **P < *0.01.* *CON* mice exposed to saline in utero, *LPS* mice exposed to inflammation in utero;

**Table 2:** The correlation between the performance in MWM and hippocampal GDNF-GFRα1 expression in F2 generation

Ages	Cognitive parameters	Groups	GDNF [r (p)]		GFRα1 [r (p)]	
			Protein	mRNA	Protein	mRNA
3 months	Swimming distance	F2-CON	− 0.247 (0.440)	− 0.453 (0.139)	− 0.047 (0.885)	− 0.423 (0.170)
		Mother-LPS	− 0.392 (0.207)	− 0.387 (0.214)	− 0.378 (0.226)	− 0.166 (0.606)
		Father-LPS	− 0.626 (0.029)*	− 0.474 (0.120)	− 0.343 (0.275)	− 0.433 (0.159)
		Parents-LPS	− 0.208 (0.517)	− 0.329 (0.296)	− 0.293 (0.356)	− 0.629 (0.029)*
	Distance percentage in target quadrant	F2-CON	0.311 (0.325)	0.533 (0.075)	0.159 (0.621)	0.378 (0.225)
		Mother-LPS	− 0.166 (0.607)	− 0.159 (0.621)	0.353 (0.260)	− 0.025 (0.940)
		Father-LPS	0.449 (0.143)	0.248 (0.438)	0.568 (0.054)	0.281 (0.376)
		Parents-LPS	0.302 (0.339)	− 0.051 (0.875)	0.254 (0.426)	0.675 (0.016)*
15 months	Swimming distance	F2-CON	− 0.125 (0.700)	− 0.468 (0.125)	− 0.387 (0.214)	− 0.026 (0.936)
		Mother-LPS	0.008 (0.980)	− 0.468 (0.125)	− 0.249 (0.435)	0.230 (0.472)
		Father-LPS	− 0.705 (0.010)*	− 0.536 (0.072)	− 0.687 (0.014)*	− 0.583 (0.047)*
		Parents-LPS	− 0.674 (0.016)*	− 0.802 (0.002)**	− 0.646 (0.023)*	− 0.639 (0.025)*
	Distance percentage in target quadrant	F2-CON	− 0.009 (0.978)	− 0.250 (0.433)	− 0.227(0.479)	− 0.038 (0.906)
		Mother-LPS	0.246 (0.441)	0.250 (0.433)	0.524 (0.081)	0.501 (0.097)
		Father-LPS	0.791 (0.002)**	0.831 (0.001)**	0.831 (0.001)**	0.939 (0.000)**
		Parents-LPS	0.853 (0.000)**	0.534 (0.073)	0.701 (0.011)*	0.863 (0.000)**

n = 6 per group

*P < *0.05*

**P < *0.01.* *F2-CON* mice whose parents were exposed to saline in utero, *Mother-LPS* mice whose mothers were exposed to inflammation in utero, *Father-LPS* mice whose fathers were exposed to inflammation in utero, *Parents-LPS* whose parents were exposed to inflammation in utero

Further, in light of the apparent correlation among pro-inflammatory cytokines and both cognition and *Gdnf* and *GFRα1* expression (see Additional file 4, 5, 6, 7 for details), we performed partial correlation analysis (controlling for IL-1β, IL-6 and TNF-α levels) as shown in Table 3 and 4. In F1 CON mice aged 3 months the percentage of distance swam was positively correlated with both GFRα1 protein (*r* = 0.772, *P* = 0.015) and mRNA (*r* = 0.695,* P* = 0.038) levels. At 15 months old, GDNF mRNA expression was negatively correlated with the distance swam in the learning phase in the LPS group (*r* =− 0.823, *P* = 0.006). In the F2 generation, there were no correlations between performance in WMW and *Gdnf and GFRα1* expression at 3 months of age. However, at 15 months of age, positive correlations between percentage of distance swam in the probe task and GFRα1 protein (*r* = 0.724, *P* = 0.028) and mRNA (*r* = 0.901,* P* = 0.001) expression were detected for the Parents-LPS group of mice. In addition, for older mice the *GFRα1 mRNA* level was negatively correlated with the distance swam in the learning phase in the Mother-LPS group (*r* = 0.705,* P* = 0.034), and positively correlated with the percentage of time spent in the target quadrant during the probe trial in the Father-LPS group (*r* = 0.722,* P* = 0.028).

**Table 3:** The partial correlation between the performance in MWM and hippocampal GDNF-GFRα1 expression in F1 generation

Cognitive parameters	Ages	Groups	GDNF [r (p)]		GFRα1 [r (p)]	
			Protein	mRNA	Protein	mRNA
Swam distance	3 months	CON	− 0.475 (0.196)	− 0.574 (0.106)	− 0.606 (0.084)	− 0.584 (0.099)
		LPS	0.236 (0.542)	− 0.510 (0.161)	0.086 (0.827)	0.665 (0.051)
	15 months	CON	− 0.435 (0.242)	− 0.447 (0.228)	− 0.579 (0.103)	− 0.043 (0.912)
		LPS	− 0.288 (0.452)	− 0.823 (0.006)**	0.149 (0.702)	0.494 (0.177)
Percentage swam distance in target quadrant	3 months	CON	0.547 (0.127)	0.663 (0.052)	0.772 (0.015)*	0.695 (0.038)*
		LPS	0.478 (0.193)	0.084 (0.830)	− 0.359 (0.343)	0.068 (0.863)
	15 months	CON	− 0.054 (0.890)	− 0.125 (0.749)	0.012 (0.976)	− 0.016(0.968)
		LPS	− 0.583 (0.099)	− 0.034 (0.930)	0.020 (0.960)	0.123 (0.753)

n = 6 per group

*P < *0.05*

**P < *0.01.* *CON* mice exposed to saline in utero, *LPS* mice exposed to inflammation in utero

**Table 4:** The partial correlation between the performance in MWM and hippocampal GDNF-GFRα1 expression in F2 generation

Cognitive parameters	Ages	Groups	GDNF [r (p)]		GFRα1 [r (p)]	
			Protein	mRNA	Protein	mRNA
Swam distance	3 months	F2-CON	− 0.273 (0.477)	− 0.502 (0.169)	− 0.498 (0.173)	− 0.562 (0.115)
		Mother-LPS	0.206 (0.594)	0.061 (0.876)	− 0.029 (0.940)	0.278 (0.468)
		Father-LPS	− 0.612 (0.080)	− 0.444 (0.232)	− 0.560 (0.117)	− 0.441 (0.234)
		Parents-LPS	0.112 (0.773)	− 0.289 (0.450)	− 0.062 (0.874)	− 0.424 (0.256)
	15 months	F2-CON	− 0.135 (0.729)	− 0.335 (0.377)	− 0.346 (0.362)	0.110 (0.778)
		Mother-LPS	− 0.631 (0.068)	− 0.645 (0.061)	− 0.586 (0.098)	−0.043 (0.913)
		Father-LPS	− 0.020 (0.959)	− 0.345 (0.363)	− 0.353 (0.352)	−0.377 (0.318)
		Parents-LPS	− 0.336 (0.377)	− 0.688 (0.041)*	− 0.436 (0.241)	−0.421 (0.259)
Percentage swam distance in target quadrant	3 months	F2-CON	0.242 (0.530)	0.563 (0.115)	0.519 (0.152)	0.535 (0.138)
		Mother-LPS	0.392 (0.297)	0.611 (0.080)	0.470 (0.202)	0.452 (0.222)
		Father-LPS	− 0.315 (0.409)	− 0.040 (0.918)	0.274 (0.475)	0.055 (0.889)
		Parents-LPS	0.157 (0.687)	− 0.090 (0.817)	0.265 (0.491)	0.320 (0.401)
	15 months	F2-CON	0.198 (0.610)	0.324 (0.395)	0.164 (0.673)	0.465 (0.207)
		Mother-LPS	− 0.073 (0.851)	0.092 (0.814)	0.428 (0.250)	0.705 (0.034)*
		Father-LPS	0.220 (0.570)	0.547 (0.127)	0.472 (0.199)	0.722 (0.028)*
		Parents-LPS	0.349 (0.358)	0.503 (0.167)	0.724 (0.028)*	0.901 (0.001)**

n = 6 per group

*P < *0.05*

**P < *0.01.* *F2-CON* mice whose parents were exposed to saline in utero, *Mother-LPS* mice whose mothers were exposed to inflammation in utero; *Father-LPS* mice whose fathers were exposed to inflammation in utero; *Parents-LPS* whose parents were exposed to inflammation in utero

## Discussion

Increasing evidence indicates that adverse maternal exposures (i.e., infectious, metabolic, or psychological stress) during gestation have a harmful impact on cognitive function of their offspring, even grandchildren [34, 35]. Our published studies showed that the effect of cognitive impairment induced by maternal exposure to LPS on the F1 generation could be observed in the F2 generation, with the major contribution of this cognitive damage in F2 offspring being derived from their fathers. Moreover, our preliminary exploration showed that this mechanism of intergenerational transmission may involve H3K9 hypermethylation and H4K12 hypoacetylation in the hippocampus [13]. However, there is a paucity of information on the specific mechanisms underlying intergenerational transmission of maternal stress. In addition, it attracted our attention that despite relevant research evidence, related studies did not focus on the interference effect of inflammatory states on the neurological mechanisms determining AACD [26, 29]. Results from the present study in mice showed that the F1 generation born from mothers exposed to LPS had poorer spatial learning and memory ability compared to control F1 mice born from mothers that received saline injections. To our surprise, similar results were observed in the Father-LPS and Parents-LPS groups, but not in the Mother-LPS group, in the F2 generation. Further, our analysis revealed lower levels of hippocampal *Gdnf* and *GFRα1* expression in the LPS-treated group in F1 generation compared to the corresponding control group. Of note, a similar finding was obtained in the F2 generation born from F1 mice exposed to LPS in utero, especially the F2 mice included in the Father-LPS and Parents-LPS groups. These results suggest that LPS-induced cognitive deficits can be transmitted into grand-offspring via paternal linage, in a manner likely associated with declined expression of *Gdnf* and *GFRα1*. In this regard, Pearson’s correlation analysis showed that expression levels of *Gdnf* and *GFRα1* in LPS-treated F1 and F2 generations at midlife (15 months of age) were negatively correlated with the swimming distance during the learning phase of the MWM task, and positively correlated with the percent distance in the target quadrant during the memory phase. However, partial correlation analyses controlling for IL-1β, IL-6, and TNF-α levels indicated only scattered correlations between *Gdnf* or *GFRα1* expression and cognitive ability. This suggested that increased levels of pro-inflammatory cytokines interfere, at least partially, with the relationship between hippocampal neurotrophins and cognitive dysfunction.

### Gestational (F0) LPS exposure accelerates age-associated cognitive decline in both F1 and F2 generations

Growing evidence indicates that maternal LPS exposure during late gestation may accelerate, starting from midlife, age-associated decline of spatial learning and memory abilities in the offspring [6, 36, 37]. Further supporting this notion, the present results showed that compared to control mice, which exhibited the expected age-related decay in MWM performance, maternal LPS exposure was associated with impaired spatial learning and memory in older (15 months old) rather than younger (3 months old) CD-1 mice.

In the present study, we demonstrate again that the effect of maternal (F0) administration on cognitive impairment in offspring can be transmitted from the F1 to the F2 generation. Specifically, and compared to age-matched controls, impaired spatial learning and memory abilities were noted in 15-months-old F2 mice in the Father-LPS and Parents-LPS groups, rather than in mice included in the Mother-LPS group. This intergenerational inheritance pattern of cognitive dysfunction through paternal linage was also reported in other stress paradigms. For instance, a study by Joushi et al. showed that cognitive impairment induced by maternal separation, a well-characterized model of early life stress, could be transmitted from F1 generation to F2 generation through the father’s lineage [37]. However, other studies suggested that emotional deficits may transmit from early-life stressed mothers, but not fathers, to their progeny [14, 38]. These discrepant findings may be due to sex differences in vulnerability to stress-induced cognitive and emotional deficits [39]. A large number of animal and human studies suggested that mechanisms for this sex bias may involve sex steroids and sex differences in the locus coeruleus and its regulation by stress [12, 40–42]. Still, further research is needed to clarify the mechanisms determining sex-specificity in intergenerational inheritance of cognitive impairment.

Unexpectedly, F2 mice born from parents which were both exposed to LPS-induced inflammation in utero did not show poorer cognitive performance than mice born from an LPS- and a saline-exposed parent. We thus inferred that the paternal and maternal effects on cognitive impairment in F2 offspring were independent, or alternatively, that a cumulative effect was not yet apparent.

### Accelerated age-associated decline in hippocampal *Gdnf*/*GFRα1* expression in F1 and F2 generation mice induced by maternal LPS exposure

Increasing evidence indicates that external factors can positively or negatively impact the expression of the *Gdnf* gene throughout the life of mammals. In this regard, food, exercise, and an enriched environment are considered as positive modulators [20, 43–45], while neuroinflammation, infections, and ageing have been proposed as negative regulators [15, 46–48]. Several studies have reported a characteristic age-associated decrease in *Gdnf* gene expression patterns in different rodent strains [49–51]. Consistent with these findings, the current results showed that ageing significantly affected the expression of both *Gdnf* and *GFRα1* genes in CD-1 mice, with lower expression detected in 15-months-old relative to 3-months-old mice.

We were interested to know whether adverse intrauterine stimuli, specifically gestational inflammation, would accelerate the age-related decline in *Gdnf* and *GFRα1* expression in the first- and second- generation offspring. Our results showed that maternal (F0) LPS exposure suppressed the expression of the two genes in the F1 and F2 generations. Importantly, and similar to our findings regarding cognitive (MWM) performance, we concluded that this intergenerational inheritance effect was mainly attributed to the paternal lineage. Specifically, lower *Gdnf* and *GFRα1* expression levels were observed in the LPS groups in F1 generation, as well as in the Father-LPS and Parents-LPS groups in F2 generation. Interestingly, F2 mice in the Parents-LPS group had lower *Gdnf* and *GFRα1* expression at the mRNA level, but not at the protein level, relative to the Father-LPS group. This implied the suppressing effect transmitted from LPS-exposed F1 mother and father on both genes’ mRNA levels may be cumulative; still, although further research is warranted, the lack of effect on the corresponding protein levels may suggest a reason why no cumulative effect was detected in the behavioural performance.

### Potential interplay between gestational inflammation and reduced *Gdnf* and* GFRα1* expression and impaired cognition in F1 and F2 generation

The neurological bases of AACD have been extensively explored, with current evidence highlighting the influence of alterations in synapsis-related proteins, neurotrophic factors, and histone modifications [6, 36, 50]. However, most studies conducted in this regard have not addressed the potential interference of gestational inflammation on manifestations of AACD in the direct or second-generation progeny. Hence, in this study we preliminarily explored the relationship between *Gdnf* and *GFRα1* expression and cognitive deficits, while controlling for interference from pro-inflammatory cytokines, in F1 and F2 offspring from LPS-treated gestating mouse dams. As expected, we observed an age-dependent increase in circulating levels of IL-1β, IL-6, and TNF-α in F1 control mice. More importantly, our data showed that compared to the latter, serum levels of the above pro-inflammatory factors were significantly increased, at both 3 and 15 months of age, in F1 mice exposed to LPS in utero. While this finding would indicate that maternal LPS exposure stimulates immune activation in the offspring, increased pro-inflammatory cytokine levels were also detected in 3-months-old mice in the F2 generation born from LPS-exposed F1 mice. This would suggest that maternal LPS exposure promotes development of a pro-inflammatory state not only in the direct progeny, but also in subsequent descendants. In agreement with these findings, administration of LPS was previously reported to result in a mild inflammatory phenotype in the F1 generation, a condition transferred in turn to the F2 generation [51].

Pearson’s correlation analysis showed that declined *Gdnf* and *GFRα1* expression correlated with accelerated AACD induced by gestational LPS exposure in F1 and F2 mice aged 15, but not 3, months. It seems reasonable to propose that decreased *Gdnf* and *GFRα*1 expression contributes to the intergenerational transmission of cognitive impairment. However, we found that the results from partial correlation analysis (controlling for pro-inflammatory cytokine expression) were not entirely consistent with those derived from the initial Pearson’s correlation analysis (see Tables 4 and 5). On the one hand, many original correlations observed in the F1 and F2 generations were no longer significant in a partial correlation analysis; for instance, *GFRα*1 expression was not related to the learning or memory performances for the LPS group in the F1 generation. On the other hand, new correlations emerged for the 3-months-old control group in the F1 generation and for the 15-months-old Mother-LPS group in the F2 generation. These results revealed that the levels of pro-inflammatory cytokines had a significant impact on the results of correlation analyses involving *Gdnf* and *GFRα1* expression and cognitive function. Combined with the other two correlation analyses, which indicated negative correlation between pro-inflammatory cytokine levels and both *Gdnf* and *GFRα*1 expression and cognitive abilities (see Additional file 4, 5, 6, 7 for details), this evidence seems to imply that increased levels of peripheral pro-inflammatory factors negatively affect, directly or indirectly, cognitive function. More generally, our findings remind us that more attention needs to be paid to the potential interference of pro-inflammatory cytokines when exploring the neurological bases of AACD.

**Table 5:** Animal data in the study

	No. of the animals	Mating paternal	Total no. of the offspring	Total female: male ratio
F0-CON	10	–	94	46:48
F0-LPS	10	–	97	50:47
F1-CON-F	10	–	93	50:43
F1-CON-M	10	F1-CON-F	91	44:47
F1-LPS-M	10	Unexposed females	99	45:54
F1-LPS-F	10	Unexposed males	102	49:53
F1-LPS-F	10	F1-LPS-M	85	40:45

## Conclusion

In conclusion, the current study further demonstrated that accelerated AACD due to exposure to gestational inflammation in the F0 generation can be transmitted from the F1 to the F2 generation, and that this effect is mainly derived from the F1 father. Moreover, for the first time, we showed that AACD possibly involves accelerated age-associated decline of *Gdnf* and *GFRα1* expression in the direct and second generation offspring resulting from maternal (F0) gestational acute inflammation. A limitation of this study was that GDNF and GFRα1 were not overexpressed or knocked down to assess the influence of these manoeuvres on learning and memory function. Hence, more research is needed to establish the precise role of GDNF-GFRα1 signalling in the intergenerational transmission of AACD.

## Materials and methods

### Animals and treatments

**Fig. 12:**
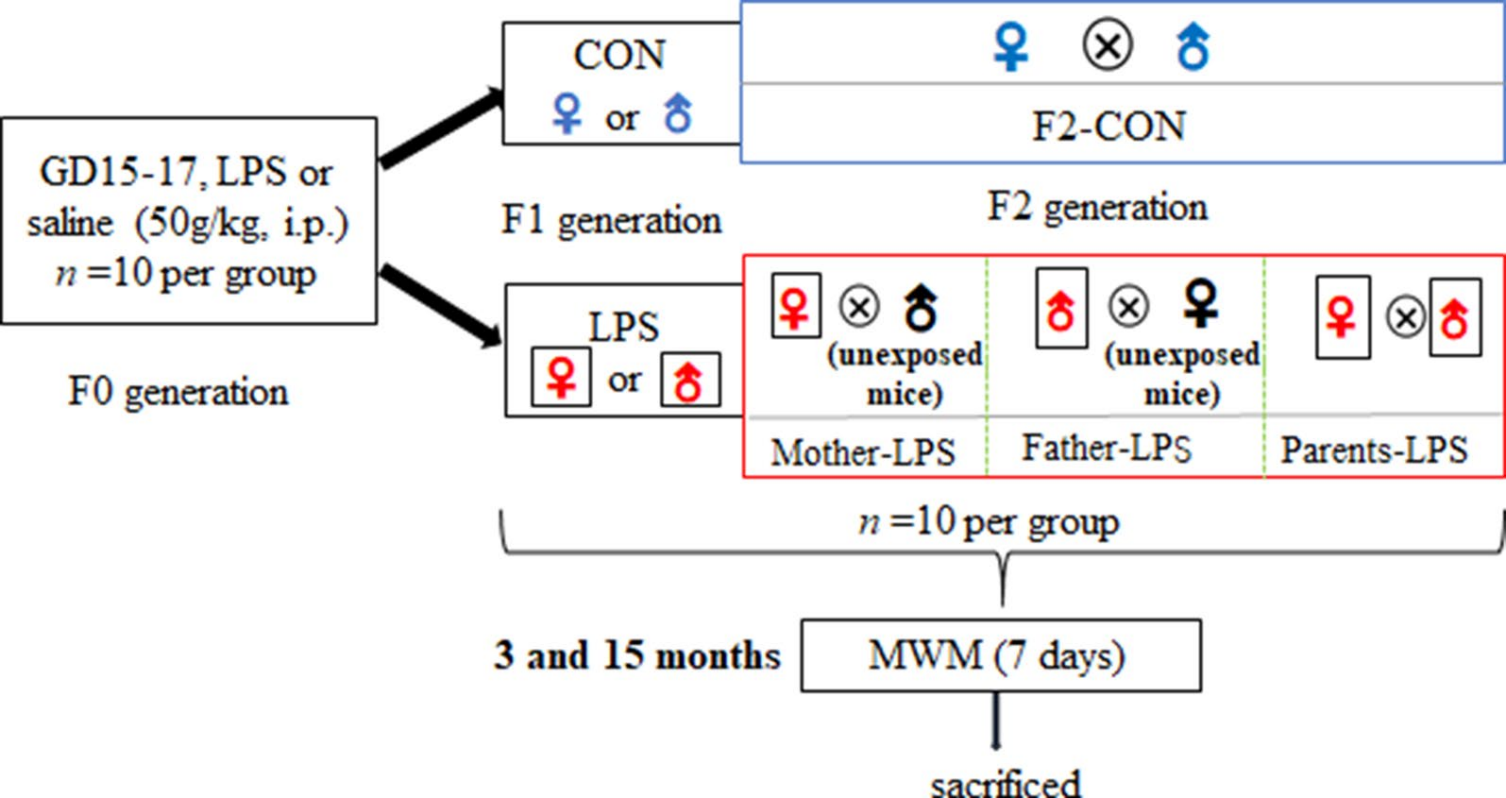
Timeline of experimental events. *CON* mice exposed to saline in utero, *LPS* mice exposed to inflammation in utero, *F2-CON* mice whose parents were exposed to saline in utero, *Mother-LPS* mice whose mothers were exposed to inflammation in utero, *Father-LPS* mice whose fathers were exposed to inflammation in utero, *Parents-LPS* mice whose parents were exposed to inflammation in utero, *MWM* Morris water maze, *LPS* lipopolysaccharide, *i.p* intraperitoneal, *gd* gestational day

CD-1 mice (6–8 weeks of age) were purchased from Beijing Vital River Laboratory Animal Technology Co. Ltd. (Beijing, China), from foundation colonies introduced by Charles River Labs, Inc. (Wilmington, MA, USA). The animals were housed in polypropylene cages (38 × 32 × 16 cm^3^) at a controlled temperature (24 ± 2 °C) and humidity (50–60%) with artificial lighting (12 h/12 h light/dark cycle; lights on at 7:00 A.M.). The animals had free access to rodent chow and filtered water. After 2-week acclimation to the living environment, males and females (1:2) were used as breeders. The day a vaginal plug appeared was designated as gestational day 0 (GD0). The pregnant mice were randomly divided into control (saline-treated) and experimental (LPS-treated) groups (n = 10 mice/group). During GDs 15–17, the LPS-treated group of mice received a daily intraperitoneal injection of LPS (50 μg/kg; Sigma, St. Louis, MO, USA), while control mice received the same volume of normal saline. The offspring (F1 generation) were kept with their mothers until postnatal day (PND) 21, before being relocated to new cages along with 4–5 sex- and parent treatment-matched animals. Subsequently, 2-months-old (PND60) male and female mice from the F1 control and LPS groups were allocated for breeding. Information on animal data is presented in Table 5. To test for effects of lineage on a second generation, the F2 generation was divided into the following four groups (n = 10 mice/group): (1) Mother-LPS (maternal lineage of LPS; LPS females mating with wild-type males); (2) Father-LPS (paternal lineage of LPS; LPS males mating with wild-type females); (3) Parents-LPS (parental lineage of LPS; LPS females mating with non-littermate LPS males); and Control (CON; control females mating with control males). F2 generation mice were similarly weaned from their mothers and housed within sex and treatment group at PND21. At the ages of 3 and 15 months, the F1 and F2 generations from the control and LPS groups were randomly selected to complete the behavioural tests. All animal experiments protocols were approved by Anhui Medical University ethics committee (LLSC20160165). The study was carried out in compliance with the guidelines for humane treatment set by the Association of Laboratory Animal Sciences and the Center for Laboratory Animal Sciences at Anhui Medical University and in accordance with ARRIVE guidelines. A schematic representation of the experimental timeline is shown in Fig. 12.

### Morris water maze

Spatial learning and memory were tested using the Morris water maze (MWM). The maze consisted of a circular black pool (150 cm in diameter and 30 cm deep), filled with clear water (21–22 ℃, depth of 25 cm) and surrounded by white curtain with three salient visual cues (circles, squares, and triangles). The surface of the pool was virtually and averagely subdivided into four quadrants. A black cylindrical escape platform (10 cm in diameter, 24 cm in height) was submerged 1 cm below the water surface near the centre of one quadrant of the maze (target quadrant I). In turn, the other three quadrants were arranged counterclockwise from the first quadrant. There was a camera system above the pool to track and record the movement of the mice. The experimental process included two phases: a positioning navigation trial (learning phase) and a probe trial (memory phase). In the learning phase, the mice were randomly dropped into the pool while facing the wall of each quadrant. Regardless of whether the mouse found the platform or not within 60 s, it was allowed to rest on the platform for 30 s at the end of the trial. The test was performed 4 times a day, with a 15-min interval between trials, for a total of 7 days. On the last day, the mice were first subjected to a positioning navigation test as before, and a probe trial was then performed 2 h after the last trial to test for long-term spatial memory. In this trial, the platform was removed and the mouse was released from the quadrant opposite to the previous platform location (target quadrant) and allowed to swim for 60 s. The average swimming latency and distance during the positioning navigation was analyzed as a measure of learning ability, while the time spent and distance travelled in the target quadrant (quadrant 1) during the probe trial were analyzed as a measure of spatial memory retention [52]. All data were recorded by ANY-maze software (Stoelting, Wood Dale, IL, USA).

### Tissue and serum preparation

To avoid potential confounding effects related to the behavioural trials, a 15-day interval from the latter was allowed before assessment of gene, protein, and cytokine expression. The mice were sacrificed by cervical dislocation, decapitated, and their brains were immediately removed and bisected on dry ice at 9:00–10:00 am. The hippocampus was stored at − 80 °C for subsequent western blotting and RT-PCR.

Whole blood was collected via the eyeball and serum was prepared by centrifuging blood samples for 5 min at 4000 rpm (4 ℃). Approximately 100 μl of serum was collected from each mouse and the serum levels of IL-1β, IL-6 and TNF-α were measured using ELISA kits according to the manufacturer’s instructions.

### Western blotting

Western blot analysis was performed as previously described [13]. The hippocampus was digested in RIPA lysis buffer and the supernatant obtained after ultrasonic crushing and centrifugation was considered as total protein. Protein concentration was measured using a BCA Protein Assay Kit. Equal amounts of protein were added to SDS-PAGE protein loading buffer (1:4), mixed, and boiled for 10 min. After cooling to room temperature, the samples and pre-stained protein markers were injected into 10% SDS-PAGE gels. Electrophoresis was performed at 80 V for 30 min and at 120 V for 1 h. Beta-actin (TA-09; Zhongshan Golden Bridge Biotechnology, Beijing, China) was used as internal standard. Isolated proteins were electrotransferred to polyvinylidene fluoride membranes (IPVH0010; Millipore, Germany). The membranes were blocked in 5% non-fat milk diluted in Tris-buffered saline with Triton X (TBST) on a platform rocker for 2 h and then incubated with primary antibodies overnight at 4 °C. The primary antibodies used were rabbit anti-GDNF (1:2000, ab176564, Abcam, Cambridge, UK) and rabbit anti-GFRα1 (1:2000, ab84106, Abcam, Cambridge, UK). After washing with TBS containing 0.1% Tween 20 (3 × 10 min), the blots were incubated with horseradish peroxidase (HRP)-conjugated anti-rabbit IgG (1:20,000, Zhongshan-Golden Bridge Biotechnology) for 2 h at room temperature. Immunoreactive protein bands were visualized using an enhanced chemiluminescent (ECL) reagent (Thermo Fisher, Waltham, MA, USA) and analyzed using ImageJ software (Media Cybernetics, Rockville, MD, USA).

### RT-PCR

To measure *Gdnf* and *GFRα1* mRNA expression by RT-PCR, left hippocampus tissue was ground to powder with liquid nitrogen and mixed with TRIzol reagent. Total RNA was obtained through standard isopropyl alcohol precipitation, ethanol washing, drying, and centrifugation. The purity and content of the extracted RNA were assessed using a spectrophotometer. Using the mRNA in total RNA (1 μg) as template, oligo (dT) and reverse transcriptase were used for reverse transcription of mRNA into cDNA under an RNAase-free environment. The resulting cDNA (1 μg) was amplified by quantitative PCR employing 5 μl of 2 × SYBR Green mixture, 1 μl of each primer (10 μM), and 2 μl of RNase-free water per sample. PCR reactions consisted of denaturation at 94 °C for 60 s, annealing at 55 °C for 60 s, and extension at 60 °C for 1 min. The 2^−△△CT^ method was applied to calculate target mRNA expression. The primer sequences are listed in Table 6.

**Table 6:** Sequences of the primers used for quantitative RT-PCR

Gene	Amplicon size(bp)	Forward primer(5ʹ → 3ʹ)	Reverse primer(5ʹ → 3ʹ)
β-actin	120	AGTGTGACGTTGACATCCGT	TGCTAGGAGCCAGAGCAGTA
GDNF	111	CACTCTGTTCTCCTCTCTCG	GTTTTCTGCAGGACAGAAGG
GFR*α*1	163	GATATATTCCGGGCAGTCCC	GGTTGCAGACTTCATTGGAC

### Statistical analysis

Results are presented as mean ± standard deviation for normally distributed data. Escape latency and distance during MWM learning trials were analyzed in SPSS 25.0 by two-way repeated measures ANOVA (days as the repeated measures factor, while age, treatment or sex as the independent factors). Parametric data were analyzed on Graph Pad Prism 8.0 using two-way ANOVA, with age, treatment or sex as independent variables. All post-hoc analyses were performed using Fisher’s least significant difference test when data variances were equal. Pearson’s and partial correlation tests were used to analyze the correlations between relative levels of hippocampal GDNF-GFRα1, MWM performance, and cytokine levels. Differences among groups were considered significant if p < 0.05.

## Supplementary Information

**Additional file 1: **The swimming velocity in F1 and F2 generation during learning phase. swimming velocity in F1 (A, B, C) and F2 generation (D, E). n = 10 per group. All data are present as the mean ± SEM. CON, mice exposed to saline in utero; LPS, mice exposed to inflammation in utero; F2-CON, the mice whose parents were exposed to saline or inflammation; Mother-LPS, mice whose mother had been exposed to inflammation in utero; Father-LPS, mice whose father had been exposed to inflammation in utero; Parents-LPS, whose parents were exposed to inflammation in utero.

**Additional file 2: **The Full-length blot for GDNF and GFRα1 in the hippocampi of F1 mice aged 3 months (3M) and 15 months (15M).

**Additional file 3: **The Full-length blot for GDNF and GFRα1 in the hippocampi of F2 mice aged 3 months (3M) and 15 months (15M).

**Additional file 4: **The correlations between the performance in Morris Water Maze and the serum levels of IL-1β, IL-6 and TNF-α in the F1 generation.

**Additional file 5: **The correlations between the performance in Morris Water Maze and the serum levels of IL-1β, IL-6 and TNF-α in the F2 generation.

**Additional file 6: **The correlations between the levels of cytokines and hippocampal GDNF-GFRα1 expression levels in F1 offspring.

**Additional file 7: **The correlations between the levels of cytokines and hippocampal GDNF-GFRα1 expression levels in F2 offspring.

## Data Availability

All data generated or analysed during this study are included in this published article [and its Additional files].

## Abbreviations

AACD: Age-associated cognition decline
ELISA: Enzyme linked immunosorbent assay
GDNF: Glial cell line‐derived neurotrophic factor
GFRα1: GDNF family receptor α1
GD: Gestational day
IL-1β: Interleukin-1β
IL-6: Interleukin-6
LPS: Lipopolysaccharide
MWM: Morris water maze
PND: Postnatal day
RT-PCR: Reverse Transcription-Polymerase Chain Reaction
TNF-α: Tumour necrosis factor-α
