# Supplementary material for: Effects of gestational inflammation on age-related cognitive decline and hippocampal Gdnf-GFRα1 levels in F1 and F2 generations of CD-1 Mice
Source: BMC Neurosci. 2023 Apr 13;24:26. doi: 10.1186/s12868-023-00793-5 (PMC10103445; doi:10.1186/s12868-023-00793-5)
Supplement: Supplementary file 1 — Additional file 1: The swimming velocity in F1 and F2 generation during learning phase. swimming velocity in F1 (A, B, C) and F2 generation (D, E). n = 10 per group. All data are present as the mean ± SEM. CON, mice exposed to saline in utero; LPS, mice exposed to inflammation in utero; F2-CON, the mice whose parents were exposed to saline or inflammation; Mother-LPS, mice whose mother had been exposed to inflammation in utero; Father-LPS, mice whose father had been exposed to inflammation in utero; Parents-LPS, whose parents were exposed to inflammation in utero. [file 12868_2023_793_MOESM1_ESM.pdf]

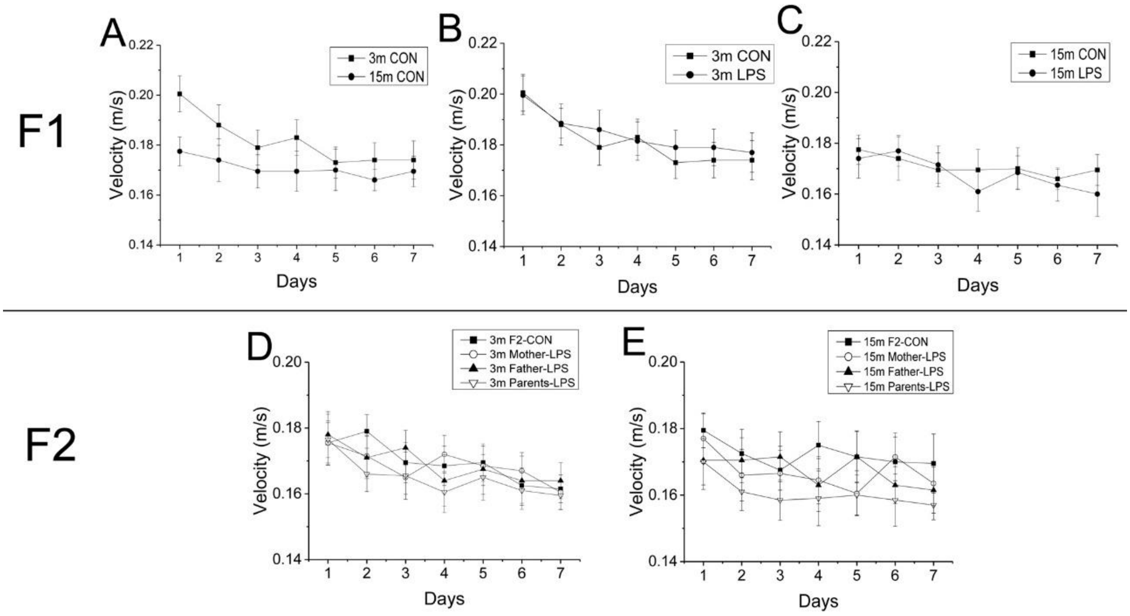

Additional file 1: the swimming velocity in F1 and F2 generation during learning phase. swimming velocity in F1 (A, B, C) and F2 generation (D, E). n = 10 per group. All data are present as the mean ± SEM. CON, mice exposed to saline in utero; LPS, mice exposed to inflammation in utero; F2-CON, the mice whose parents were exposed to saline or inflammation; Mother-LPS, mice whose mother had been exposed to inflammation in utero; Father-LPS, mice whose father had been exposed to inflammation in utero; Parents-LPS, whose parents were exposed to inflammation in utero.
