# Supplementary material for: Effects of gestational inflammation on age-related cognitive decline and hippocampal Gdnf-GFRα1 levels in F1 and F2 generations of CD-1 Mice
Source: BMC Neurosci. 2023 Apr 13;24:26. doi: 10.1186/s12868-023-00793-5 (PMC10103445; doi:10.1186/s12868-023-00793-5)
Supplement: Supplementary file 3 — Additional file 3: The Full-length blot for GDNF and GFRα1 in the hippocampi of F2 mice aged 3 months (3M) and 15 months (15M). [file 12868_2023_793_MOESM3_ESM.pdf]

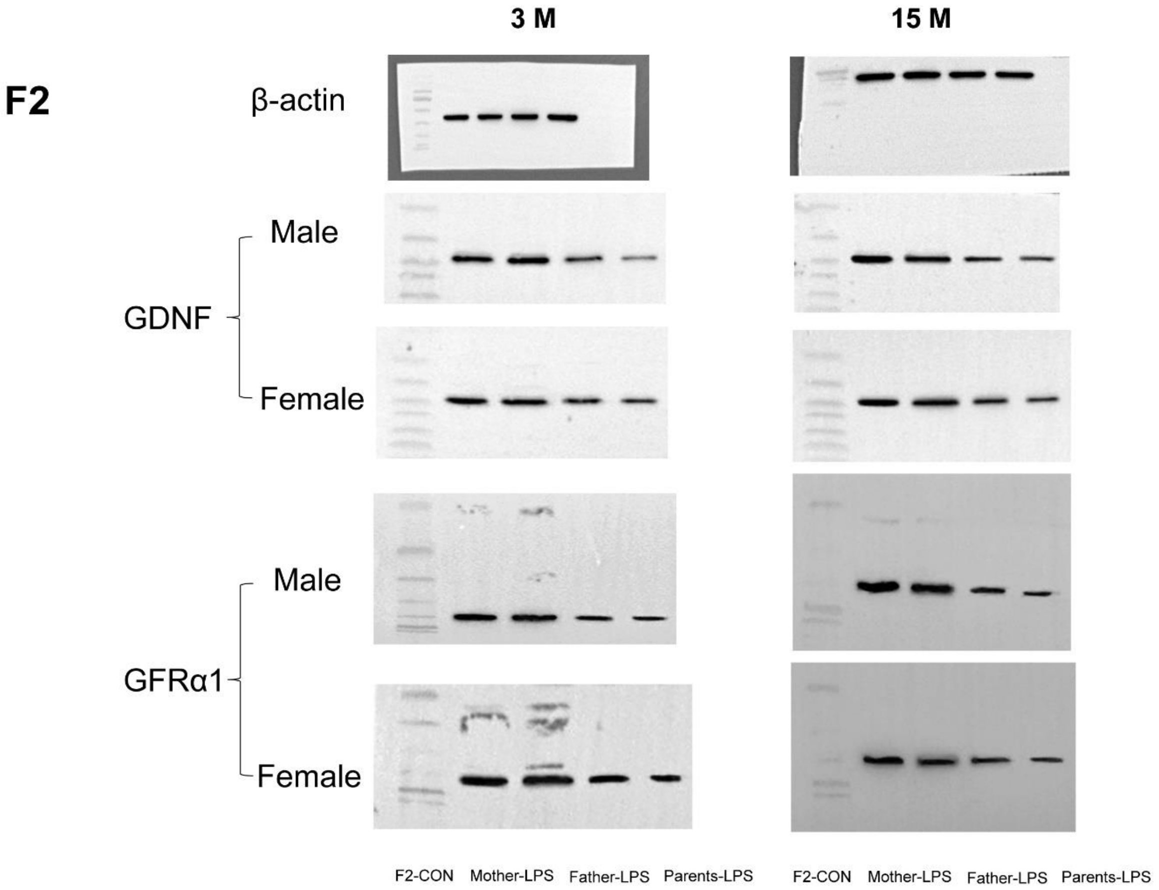

Additional file 3: the Full-length blot for GDNF and GFRα1 in the hippocampi of F2 mice aged 3 months (3M) and 15 months (15M).
