# Supplementary material for: Effects of gestational inflammation on age-related cognitive decline and hippocampal Gdnf-GFRα1 levels in F1 and F2 generations of CD-1 Mice
Source: BMC Neurosci. 2023 Apr 13;24:26. doi: 10.1186/s12868-023-00793-5 (PMC10103445; doi:10.1186/s12868-023-00793-5)
Supplement: Supplementary file 4 — Additional file 4: The correlations between the performance in Morris Water Maze and the serum levels of IL-1β, IL-6 and TNF-α in the F1 generation. [file 12868_2023_793_MOESM4_ESM.docx]

Additional file 4 The correlations between the performance in Morris Water Maze and the serum levels of IL-1β, IL-6 and TNF-α in the F1 generation.

| Ages | Cognitive parameters | Groups | IL-1β [r (p)] | IL-6 [r (p)] | TNF-α [r (p)] |
| --- | --- | --- | --- | --- | --- |
| 3 months | Swam distance | CON | 0.121 (0.611) | 0.141 (0.553) | 0.395 (0.085) |
|  |  | LPS | 0.268 (0.253) | 0.628 (0.003)** | 0.070 (0.768) |
|  | Percentage swam distance in target quadrant | CON | 0.114 (0.632) | -0.421 (0.065) | -0.027 (0.909) |
|  |  | LPS | -0.336 (0.147) | -0.419 (0.066) | 0.064 (0.790) |
| 15 months | Swam distance | CON | 0.212 (0.369) | 0.361 (0.118) | 0.395 (0.085) |
|  |  | LPS | -0.186 (0.432) | -0.409 (0.073) | -0.260 (0.268) |
|  | Percentage swam distance in target quadrant | CON | 0.443 (0.050) | 0.725 (0.000)** | 0.264 (0.261) |
|  |  | LPS | -0.530 (0.016)* | -0.685 (0.001)** | -0.380 (0.098) |

n = 10 per group. **P < 0.05, **P < 0.01.* CON, mice exposed to saline in utero; LPS, mice exposed to inflammation in utero;
