# Supplementary material for: Effects of gestational inflammation on age-related cognitive decline and hippocampal Gdnf-GFRα1 levels in F1 and F2 generations of CD-1 Mice
Source: BMC Neurosci. 2023 Apr 13;24:26. doi: 10.1186/s12868-023-00793-5 (PMC10103445; doi:10.1186/s12868-023-00793-5)
Supplement: Supplementary file 5 — Additional file 5: The correlations between the performance in Morris Water Maze and the serum levels of IL-1β, IL-6 and TNF-α in the F2 generation. [file 12868_2023_793_MOESM5_ESM.pdf]

Additional file 5 The correlations between the performance in Morris Water Maze and the serum levels of IL-1β, IL-6 and TNF-α in the F2 generation.

**Ages**	**Cognitive phase**	**Groups**	**IL-1β** [r (p)]	**IL-6** [r (p)]	**TNF-α** [r (p)]
3 months	Swam distance	F2CON	-0.040 (0.866)	-0.140 (0.556)	0.061 (0.798)
		Mother-LPS	-0.245 (0.297)	0.391 (0.088)	-0.067 (0.778)
		Father-LPS	0.359 (0.120)	0.575 (0.008)**	0.392 (0.087)
		Parents-LPS	-0.259 (0.271)	0.050 (0.833)	-0.046 (0.846)
	Percentage swam distance in target quadrant	F2CON	-0.093 (0.697)	-0.137 (0.564)	-0.104 (0.663)
		Mother-LPS	0.147 (0.535)	0.230 (0.329)	-0.067 (0.780)
		Father-LPS	-0.234 (0.320)	-0.635 (0.003)**	-0.275 (0.241)
		Parents-LPS	-0.394 (0.086)	-0.374 (0.104)	-0.488 (0.029)*
15 months	Swam distance	F2CON	0.296 (0.206)	0.342 (0.141)	0.427 (0.061)
		Mother-LPS	0.238 (0.313)	0.196 (0.407)	0.023 (0.923)
		Father-LPS	0.141 (0.553)	-0.022 (0.926)	0.264 (0.261)
		Parents-LPS	0.145 (0.543)	0.487 (0.029)*	0.488 (0.029)*
	Percentage swam distance in target quadrant	F2CON	-0.275 (0.241)	-0.485 (0.030)*	-0.430 (0.059)
		Mother-LPS	-0.150 (0.528)	-0.204 (0.388)	-0.055 (0.817)
		Father-LPS	0.267 (0.255)	-0.278 (0.235)	-0.327 (0.160)
		Parents-LPS-	-0.186 (0.432)	-0.648 (0.002)**	-0.442 (0.051)

n = 10 per group. **$P < 0.05$*, ***$P < 0.01$*. F2-CON, mice whose parents were exposed to saline in utero; Mother-LPS, mice whose mothers were exposed to inflammation in utero; Father-LPS, mice whose fathers were exposed to inflammation in utero; Parents-LPS, whose parents were exposed to inflammation in utero.
