# Supplementary material for: Effects of gestational inflammation on age-related cognitive decline and hippocampal Gdnf-GFRα1 levels in F1 and F2 generations of CD-1 Mice
Source: BMC Neurosci. 2023 Apr 13;24:26. doi: 10.1186/s12868-023-00793-5 (PMC10103445; doi:10.1186/s12868-023-00793-5)
Supplement: Supplementary file 6 — Additional file 6: The correlations between the levels of cytokines and hippocampal GDNF-GFRα1 expression levels in F1 offspring. [file 12868_2023_793_MOESM6_ESM.pdf]

Additional file 6 The correlations between the levels of cytokines and hippocampal GDNF-GFRα1 expression levels in F1 offspring

Ages	cytokines	Groups	GDNF [r (p)]		GFRα1 [r (p)]	
			Protein	mRNA	Protein	mRNA
3 months	IL-1β	CON	0.261 (0.413)	0.220 (0.491)	0.134 (0.677)	0.215 (0.502)
		LPS	-0.229 (0.474)	-0.378 (0.226)	-0.404 (0.193)	-0.462 (0.130)
	IL-6	CON	-0.282 (0.375)	-0.063 (0.846)	0.020 (0.950)	0.004 (0.991)
		LPS	0.141 (0.662)	-0.342 (0.277)	0.167 (0.603)	-0.285 (0.369)
	TNF-α	CON	-0.206 (0.522)	-0.256 (0.422)	-0.548 (0.065)	-0.458 (0.134)
		LPS	-0.657 (0.020)*	0.181 (0.574)	-0.314 (0.320)	-0.074 (0.820)
15 months	IL-1β	CON	-0.086 (0.790)	-0.005 (0.988)	-0.094 (0.771)	0.320 (0.310)
		LPS	-0.453 (0.139)	-0.521 (0.082)	-0.581 (0.048)*	-0.556 (0.060)
	IL-6	CON	-0.478 (0.116)	-0.320 (0.311)	-0.216 (0.501)	-0.323 (0.306)
		LPS	-0.244 (0.445)	-0.520 (0.083)	-0.370 (0.236)	-0.366 (0.242)
	TNF-α	CON	0.030 (0.926)	-0.114 (0.724)	-0.185 (0.564)	-0.078 (0.811)
		LPS	-0.928 (0.000)**	-0.893 (0.000)**	-0.766 (0.004)**	-0.828 (0.001)**

n = 6 per group. * $P < 0.05$, ** $P < 0.01$. CON, mice exposed to saline in utero; LPS, mice exposed to inflammation in utero;
