# Supplementary material for: Effects of gestational inflammation on age-related cognitive decline and hippocampal Gdnf-GFRα1 levels in F1 and F2 generations of CD-1 Mice
Source: BMC Neurosci. 2023 Apr 13;24:26. doi: 10.1186/s12868-023-00793-5 (PMC10103445; doi:10.1186/s12868-023-00793-5)
Supplement: Supplementary file 7 — Additional file 7: The correlations between the levels of cytokines and hippocampal GDNF-GFRα1 expression levels in F2 offspring. [file 12868_2023_793_MOESM7_ESM.pdf]

Additional file 7 The correlations between the levels of cytokines and hippocampal GDNF-GFRα1 expression levels in F2 offspring

Ages	cytokines	Groups	GDNF [r (p)]		GFRα1 [r (p)]	
			Protein	mRNA	Protein	mRNA
3 months	IL-1β	F2-CON	-0.310 (0.326)	-0.054 (0.868)	-0.039 (0.905)	0.456 (0.136)
		Mother-LPS	0.154 (0.633)	-0.040 (0.903)	0.120 (0.711)	0.458 (0.134)
		Father-LPS	0.031 (0.924)	0.360 (0.250)	-0.278 (0.382)	0.256 (0.421)
		Parents-LPS	-0.033 (0.919)	0-.068 (0.834)	0.247 (0.438)	0.129 (0.689)
	IL-6	F2-CON	-0.091 (0.778)	0.263 (0.408)	0.191 (0.553)	0.152 (0.637)
		Mother-LPS	-0.201 (0.531)	0.090 (0.781)	0.413 (0.183)	0.321 (0.309)
		Father-LPS	0.361 (0.250)	0.039 (0.903)	0.300 (0.343)	0.548 (0.065)
		Parents-LPS	0.202 (0.528)	-0.293 (0.355)	-0.202 (0.529)	-0.419 (0.175)
	TNF-α	F2-CON	-0.363 (0.246)	0.083 (0.798)	0.082 (0.801)	0.320 (0.310)
		Mother-LPS	-0.363 (0.247)	-0.396 (0.202)	0.033 (0.918)	0.324 (0.305)
		Father-LPS	-0.322 (0.307)	-0.273 (0.390)	-0.727 (0.007)**	-0.150 (0.642)
		Parents-LPS	-0.527 (0.078)	0.286 (0.367)	-0.310 (0.327)	-0.026 (0.936)
15 months	IL-1β	F2-CON	0.033 (0.918)	-0.342 (0.276)	-0.160 (0.620)	-0.234 (0.463)
		Mother-LPS	0.015 (0.963)	-0.248 (0.437)	0.268 (0.400)	-0.095 (0.770)
		Father-LPS	-0.097 (0.765)	-0.057 (0.860)	-0.021 (0.948)	-0.025(0.940)
		Parents-LPS	-0.032 (0.921)	0.086 (0.790)	0.079 (0.807)	-0.156 (0.629)
	IL-6	F2-CON	-0.217 (0.498)	-0.217 (0.498)	-0.054 (0.868)	-0.379 (0.225)
		Mother-LPS	-0.116 (0.719)	0.141 (0.661)	0.044 (0.892)	-0.073 (0.821)
		Father-LPS	-0.548 (0.065)	-0.435 (0.157)	-0.287 (0.367)	-0.504 (0.095)
		Parents-LPS	0.077 (0.811)	0.069 (0.831)	0.039 (0.904)	-0.103 (0.749)
	TNF-α	F2-CON	-0.128 (0.691)	-0.323 (0.306)	-0.325 (0.302)	-0.551 (0.063)
		Mother-LPS	-0.823 (0.001)**	-0.312 (0.323)	-0.624 (0.030)*	-0.709 (0.010)**
		Father-LPS	-0.270 (0.397)	-0.120 (0.710)	-0.543 (0.068)	-0.342 (0.276)
		Parents-LPS	-0.518 (0.085)	-0.523 (0.081)	-0.485 (0.110)	-0.672 (0.017)*

n = 6 per group. * $P < 0.05$, ** $P < 0.01$. F2-CON, mice whose parents were exposed to saline in utero; Mother-LPS, mice whose mothers were exposed to inflammation in utero; Father-LPS, mice whose fathers were exposed to inflammation in utero; Parents-LPS, whose parents were exposed to inflammation in utero.
